# Intra-patient neuraminidase mutations in avian H5N1 influenza virus reduce sialidase activity to complement weaker hemagglutinin binding and facilitate human infection

**DOI:** 10.1371/journal.ppat.1013863

**Published:** 2026-01-23

**Authors:** Yohei Watanabe, Madiha S. Ibrahim, Yasuha Arai, Daisuke Kuroda, Emad M. Elgendy, Shin-ichi Nakakita, Yohei Takeda, Vuong Nghia Bui, Takao Ono, Shota Ushiba, Tomo Daidoji, Nongluk Sriwilaijaroen, Haruko Ogawa, Kazuhiko Matsumoto, Yasuo Suzuki, Takaaki Nakaya

**Affiliations:** 1 Department of Virology, The JIKEI University School of Medicine, Tokyo, Japan; 2 Department of Infectious Diseases, Graduate School of Medical Science, Kyoto Prefectural University of Medicine, Kyoto, Japan; 3 Department of Virology, Faculty of Veterinary Medicine, Damanhour University, Damanhour, Egypt; 4 Department of Biosciences, College of Humanities and Sciences, Nihon University, Tokyo, Japan; 5 Division of Functional Glycomics, Kagawa University, Kagawa, Japan; 6 Department of Veterinary Medicine, Obihiro University of Agriculture and Veterinary Medicine, Hokkaido, Japan; 7 Research Center for Global Agromedicine, Obihiro University of Agriculture and Veterinary Medicine, Hokkaido, Japan; 8 Virology Department, National Institute of Veterinary Research, Hanoi, Vietnam; 9 Department of Mechanical Science and Bioengineering, Graduate School of Engineering Science, The University of Osaka, Osaka, Japan; 10 Murata Manufacturing Co., Ltd, Kyoto, Japan; 11 Department of Pathobiology, School of Veterinary Medicine, Rakuno Gakuen University, Hokkaido, Japan; 12 Department of Preclinical Sciences, Faculty of Medicine, Thammasat University, Pathumthani, Thailand; 13 Department of Biochemistry, School of Pharmaceutical Sciences, University of Shizuoka, Japan; 14 SANKEN, The University of Osaka, Osaka, Japan; Emory University School of Medicine, UNITED STATES OF AMERICA

## Abstract

Clade 2.2 H5N1 influenza viruses have caused an unusually high number of human infections, providing a unique opportunity to investigate early molecular steps associated with host adaptation. Although most work has focused on hemagglutinin (HA), the contribution of neuraminidase (NA) to these early adaptive events has remained unclear. By analyzing publicly available sequences from clade 2.2-infected patients, we identified 20 NA mutations and compared their phenotypes to 20 mutations acquired during diversification in primary human airway cells under drug-free conditions. Most patient-derived NA mutations resulted in modest reductions in sialidase activity, keeping activity within a functional range that supported improved replication in α2,6 sialylglycan (α2,6 Sia)-dominant environments, whereas excessive reduction impaired fitness. Notably, the phenotypes of culture-selected and patient-derived mutations were highly concordant, suggesting that these NA changes arose through natural selection rather than antiviral pressure. Re-analysis of patient sequences further revealed that many adaptive NA mutations co-occur with HA mutations that confer only weak, partial α2,6 Sia binding. Using reverse genetics, we found that such naturally occurring HA/NA mutation pairs acted cooperatively in a receptor–context-dependent manner to support α2,6-associated replication relative to HA-only mutants, placing these variants within a constrained “early-adaptation space” characterized by limited α2,6 engagement and moderately reduced NA activity. Together, these findings indicate that early human adaptation of clade 2.2 H5N1 involves not only HA and PB2, but also incremental, cooperative tuning of NA function. Monitoring coordinated HA–NA evolution may therefore improve risk assessment frameworks for zoonotic influenza viruses poised at early stages of human host adaptation.

## Author summary

Avian influenza viruses rarely infect humans because they bind poorly to receptors in the human airway. However, clade 2.2 H5N1 has caused an unusually high number of human infections, providing a unique opportunity to investigate how these viruses begin adapting to human hosts. While most research has focused on hemagglutinin (HA), which mediates host receptor binding, less is understood about the role of neuraminidase (NA), the other viral surface glycoprotein. In this study, we analyzed NA mutations detected in viruses isolated from clade 2.2-infected patients and compared them with mutations that emerged during virus replication in human airway cell cultures. We found that these NA mutations modestly adjusted enzyme activity in ways that supported replication under receptor conditions resembling those in the human airway. Importantly, many of the NA mutations identified in patients occurred alongside HA mutations previously shown to confer only weak increases in binding to human-type receptors. Using reverse-genetics-derived viruses, we found that these naturally co-occurring HA and NA mutations exhibited modest cooperative effects under human airway-like receptor conditions. These findings suggest that coordinated, incremental changes in both genes may contribute to the earliest stages of viral adaptation to humans. Together, this work highlights NA as an important—but previously underappreciated—component of early human adaptation in avian influenza viruses. Monitoring these coordinated genetic changes may strengthen genomic surveillance and pandemic preparedness.

## Introduction

The highly pathogenic avian influenza virus subtype H5N1 is currently prevalent worldwide, posing severe burdens on human public health systems. The H5N1 virus appeared in southern China in 1996, causing outbreaks in bird species and sporadic human infections, mainly in Egypt and Southeast Asia after expanding across the Eurasian continent via bird migration since 2005 [1,2]. In particular, H5N1 clade 2.2 was characteristic of single-clade epidemics in Egypt from 2006 to 2017 [3,4] causing a total of 359 human infections, the most for any country according to WHO (https://www.who.int). In contrast, in Southeast Asian countries such as Vietnam and Indonesia, multiple clades (e.g., 1.1, 2.3, and 7) caused a total of 412 cases of human infection [5,6]. Epidemics of these H5N1 clades have become sporadic since 2017 [7,8], coinciding with the emergence of other avian influenza virus subtypes such as H9N2 [9] and H5N8 [10]. However, the descendant H5N1 clade 2.3.4.4b emerged in Asia beginning in 2020, and has spread globally [11,12]. This clade is currently the most devastating zoonotic influenza virus and has caused numerous outbreaks in birds and several human infections worldwide.

Influenza A viruses express two surface proteins, hemagglutinin (HA) and neuraminidase (NA) [13]. HA functions to attach virions to host cells by binding to sialylglycans (Sia) on the target cell surface, whereas NA acts to dissociate virions from this receptor via sialidase activity. NA is thought to be necessary for the release of progeny viruses from infected cells, but recent studies noted that NA also influences initial binding and rolling of virus particles over the cell surface [14,15].

HA distinguishes between sialic acid-galactose linkages at the end of Sia [13]. Avian influenza viruses recognize the α2,3 linkage (α2,3 Sia), which is abundantly expressed in the avian intestine, whereas seasonal influenza viruses strongly bind to the α2,6 linkage (α2,6 Sia), strongly expressed in the human upper airway. For influenza viruses to cause a pandemic, receptor tropism has to change from avian-type to human-type [16]. To date, numerous H5N1 HA mutations that increase α2,6 Sia binding affinity have been reported [17–22], but this binding was still much weaker than that of seasonal influenza viruses [22–25]. Thus, avian influenza viruses would need to accumulate multiple HA mutations over time to undergo gradual fine tuning to fully switch receptor tropisms, as illustrated by the 1968 H3N2 pandemic [26].

A balance between the opposing functions of HA and NA is vital for successful viral infectivity [14,27–32]. When avian influenza viruses with a typical bird-like tropism reach the upper airway in humans, they are exposed to an environment in which they would need to infect host cells through the inherently weak α2,6 Sia binding affinity. However, successful infection would need a change of the viral HA-NA balance for Sia. Thus, we hypothesized that adaptive mutations both in HA and NA that shift their functional balance would occur during avian influenza virus infection of humans. The human-adaptive mutations of influenza viruses have so far been analyzed mainly in HA and polymerase complexes [18,19,21,33,34]. Conversely, NA mutations have been analyzed exclusively for escape from NA inhibitors (NAIs) [35,36], with one recent study reporting adaptive NA mutations naturally selected in avian influenza virus-infected patients [37].

In Egypt, single clade 2.2 epidemics have caused human infections accumulating over time. This allowed us to search for adaptive mutations that may occur rarely in patients but would be detectable with high accuracy due to the prevalence of homogeneous single-clade viral gene sequences. This was not possible with cases from another hot-spot of H5N1 human infection, Southeast Asia, where multiple clades have circulated [5,6].

We have previously characterized adaptive HA mutations in clade 2.2 virus-infected patients by database searches [18]. Here, we aimed to characterize the NA mutations that were selected for in human infections. We identified a total of 20 adaptive NA mutations in clade 2.2-infected patients. In parallel, we identified 20 intra-cellular NA mutations occurring as a result of clade 2.2 diversification in primary human cells cultures in vitro. We then characterized the viruses with adaptive NA mutations by carefully comparing the phenotypic effects of the two sets of NA mutations. Our data reveal a mechanism involving complementary NA mutations in H5N1 influenza virus variants in patients which compensate for the weaker HA Sia binding in the human airway. These might also be applicable to other influenza virus adaptations to humans.

## Results

### Identification of human-adaptive NA mutations acquired by the H5N1 virus in vivo and in vitro

**Fig 1:**
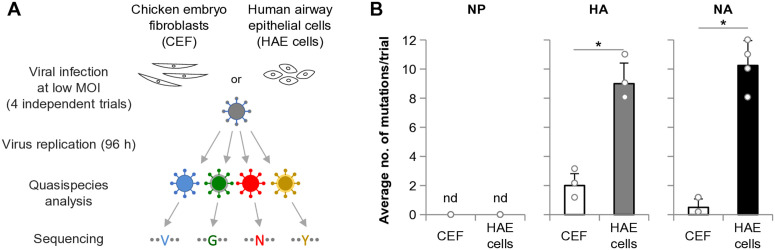
In vitro diversification of clade 2.2 NA during virus replication. (A) Schematic overview of the in vitro diversification experiment. Primary HAE cells and CEFs as controls were infected with recombinant clade 2.2 EG/D1 virus with high genetic homogeneity at MOIs of 0.1 (HAE) and 0.01 (CEF). After 96 hpi, quasispecies diversity in progeny viruses was assessed by sequencing, using a 3% variant-calling threshold. Four independent experiments were conducted. (B) Average number of amino acid substitutions detected in NP (left), HA (middle), and NA (right). Each data point represents the mean ± SD of four independent experiments. *P < 0.01. “nd” indicates not detected.

We first investigated whether the H5N1 virus NA gene can potentially acquire adaptive mutations during virus replication in humans. To this end, we analyzed clade 2.2 genetic diversification during single infection in primary human airway epithelial (HAE) cells and chicken embryo fibroblasts (CEFs) as a control (Fig 1A). The two cell strains were infected with recombinant clade 2.2 virus with a high degree of genetic homogeneity. The progeny viruses were analyzed 96 hours post-infection (hpi), as reported previously [19]. No amino acid mutations were observed in the internal viral protein NP in these cells (Fig 1B). On the other hand, approximately the same number of mutations were detected in both the HA and NA in HAE cells, but very few mutations were observed in CEFs, indicating selective pressure specific to human cells. These results suggested that human-adaptive mutations occur in the NA as frequently as in the HA during clade 2.2 replication in human airway cells.

**Fig 2:**
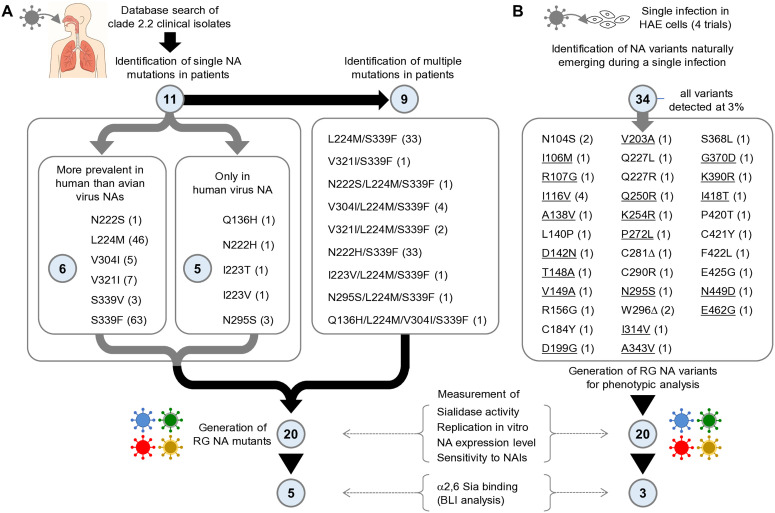
Schematic workflow for identifying and functionally characterizing human-adaptive NA mutations in clade 2.2 viruses. (A) Intra-patient NA mutations. Database analysis of H5N1 clade 2.2 clinical isolates identified 11 single NA mutations, grouped into two categories: (i) mutations detected more frequently in human-derived than avian-derived viruses (6 mutations), and (ii) mutations detected exclusively in human-derived viruses (5 mutations). Numbers in parentheses indicate the number of patients in whom each mutation was detected. A search for co-occurring changes revealed 9 multiple-mutation combinations, yielding 20 total patient-associated NA mutations. Recombinant EG/D1 viruses carrying each mutation were generated for phenotypic evaluation. (B) Intra-cellular NA mutations. To identify NA mutations that naturally emerge during infection of human airway epithelium, primary HAE cells were infected once with genetically homogeneous EG/D1 virus at a low MOI, and progeny virus quasispecies were analyzed after a single round of multicycle replication (Fig 1). Importantly, no serial passaging or iterative selection procedures were performed, ensuring that mutations reflected naturally arising diversity within a single infection cycle rather than laboratory-driven adaptation. Across four independent experiments, 34 NA mutations were detected as ≥3% minor variants. Numbers in parentheses indicate detection frequency among the four trials. Of these, 20 NA mutations (underlined) were successfully incorporated into recombinant EG/D1 viruses for evaluation of sialidase activity, Sia-dependent replication, NA protein expression, and susceptibility to NA inhibitors. Based on phenotypic characteristics, 8 mutants (5 intra-patient, 3 intra-cellular; N295S found in both groups) were selected for α2,6 Sia binding analysis by biolayer interferometry. The image was generated using Open AI (ChatGPT) and edited manually (licensed under CC BY 4.0).

We next sought to identify the NA mutations that were selected for in H5N1 virus-infected patients. To this end, we conducted a database search for the clade 2.2 virus gene sequence registered in the GISAID database (https://gisaid.org). This yielded 94 NA gene sequences derived from Egyptian patients (2006–2012) and 343 NA gene sequences derived from birds from the same region over the same period. We then searched for NA amino acid mutations characteristic of human-derived viruses by comparing each of the gene sequences with the consensus sequence determined by aligning all NA sequences. As a result, we identified 11 individual NA mutations in the infected patients (see Fig 2A for a flow-chart of identification and characterization of intra-patient NA mutations). These single NA mutations were classified into two categories. The first comprised NA mutations that were detected more frequently in human viruses than avian viruses (6 mutations), which had presumably emerged in the field and were subsequently transmitted from birds to humans with high efficacy. The second comprised NA mutations that were detected only in the human-derived viruses, but not in the avian viruses (5 mutations), which presumably emerged within the infected individuals. We additionally searched for combinations of these single NA mutations in all infected patients, and found 9 instances where multiple mutations co-occurred. A/duck/Egypt/D1Br/2007 (EG/D1), one of the parental clade 2.2 strains [20], was used as the reverse-genetics (RG) backbone to generate recombinant viruses carrying intra-patient NA mutations. In total, 20 recombinant EG/D1 viruses were constructed: 11 bearing single NA mutations detected in patients, and 9 bearing naturally occurring combinations of these mutations. All viruses were successfully rescued by RG.

Clade 2.2 virus-infected patients had often been treated with NAIs in Egypt [4]. This raised the possibility that the identified intra-patient NA mutations potentially included NAI escape mutations, although their overall occurrence in the H5N1 virus is lower than in seasonal influenza viruses [35]. We therefore characterized the intra-cellular NA mutations appearing in HAE-infected cells (Fig 1) and compared the phenotypic changes mediated by intra-patient NA mutants and intra-cellular NA mutants (see Fig 2B for a flow-chart of identification and characterization of intra-cellular NA mutations). Thirty-four single NA mutations were detected from 4 independent infections, with the mutations all existing as minor single variants at a frequency of 3%. We then generated recombinant EG/D1 viruses having each one of the intra-cellular NA mutations and succeeded in creating 20 replicative mutants. Of note was the N295S mutation, which was detected in both intra-patient and intra-cellular NA mutations. In total, 39 adaptive NA mutant viruses were analyzed in further studies.

### Adaptive NA mutant viruses have slightly reduced sialidase activity

Because influenza viruses attach, enter, and disseminate as intact particles, we first quantified virion-level (“net”) sialidase activity after normalizing purified virions by infectious particle number (focus-forming units; FFU). This approach measures the overall NA function per infectious virion—an essential determinant of HA–NA functional balance during entry and release—rather than the catalytic efficiency per NA molecule. We therefore considered FFU-normalized NA activity as the most biologically relevant readout to capture how each NA mutation alters the net receptor-dissociating capacity of virus particles. EG/D1 HA has an inherently strong binding affinity for α2,3 Sia, but only a weak affinity for α2,6 Sia [20], indicative of a typical avian tropism. We also included H275Y as a reference NA mutation, which significantly reduced H5N1 virus sialidase activity and serves as a representative example of resistance against oseltamivir and peramivir [28].

In addition to this virion-level measurement, we also assessed “NA amount–normalized (intrinsic) NA activity,” defined as sialidase activity normalized by NA protein abundance determined by Western blotting. This complementary metric reflects the intrinsic catalytic efficiency of NA independently of its incorporation level into virions. Throughout this study, we distinguish between “FFU-normalized (virion-level, net) NA activity” and “NA amount–normalized (intrinsic) NA activity,” using each parameter for the appropriate biological question (results shown in S4 and S5 Figs).

**Fig 3:**
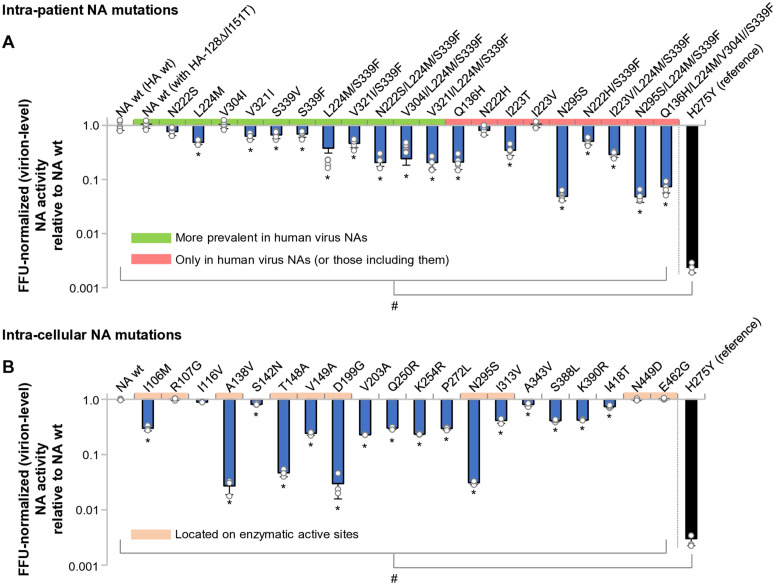
Effects of human-adaptive NA mutations on FFU-normalized (virion-level, net) sialidase activity of H5N1 virus particles. (A) Intra-patient NA mutations and (B) intra-cellular NA mutations were evaluated using a chemiluminescent sialidase assay with NA-XTD substrate. To measure virion-level (“net”) NA activity, purified virus preparations were normalized by FFU prior to assay. Sialidase activity values are expressed relative to the FFU-normalized activity of NA-wt virus. The H275Y mutant was included as a reference for markedly reduced NA function. Data represent mean ± SD from three independent experiments. *P < 0.01. # indicates that the H275Y mutant differed significantly from all other NA mutants as well as NA-wt (*P* < 0.01). Statistical analysis was performed using one-way ANOVA with Tukey’s multiple comparison test. Note: This figure reports FFU-normalized (virion-level, net) NA activity; analyses of NA amount–normalized (intrinsic) NA activity are presented separately in S5 Fig.

Most intra-patient-derived NA mutant viruses exerted reduced FFU-normalized (virion-level, net) NA activity relative to the NA wild-type (wt) (Fig 3A). Among them, the N295S and the N295/L224M/S339F viruses exerted the weakest FFU-normalized NA activity, at about one-fifth of the activity of the NA-wt. Nevertheless, the sialidase activity of all the NA mutants was still >10-fold higher than that of the H275Y virus. Similarly, most intra-cellular-derived NA mutants had reduced FFU-normalized NA activity relative to NA-wt (Fig 3B). A138V and D199G viruses had reduced FFU-normalized NA activity to an extent similar to the N295S virus, but again, all viruses still had activity >10-fold that of the H275Y virus. These results showed that both the intra-patient and intra-cellular NA mutants had modestly reduced FFU-normalized NA activity.

### Adaptive NA mutations enhance virus yields in an α2,6 Sia-dependent manner

We next evaluated Sia-dependent propagation of the NA mutant viruses using four different cell strains with distinct Sia expression patterns. Because the interpretation of Sia-dependent replication critically depends on the relative availability of α2,3- and α2,6- Sias on each cell line, we first confirmed their Sia expression profiles by fluorescent lectin staining (S1 Fig). DF-1 cells showed strong Maackia amurensis lectin I (MAL-I) staining with negligible Sambucus nigra lectin (SNA) signal, indicating predominant α2,3 Sia expression. MDCK cells expressed both α2,3 and α2,6 Sias but remained α2,3-dominant. In contrast, 1A5 cells displayed robust α2,6 Sia expression with detectable, but weaker α2,3 Sia signals. These expression patterns were consistent with previous reports [38–41]. Importantly, α2,3-sialidase-treated 1A5 cells exhibited nearly complete loss of α2,3 Sia immediately after treatment and remained undetectable for at least 13 h. The α2,3 sialidase-treated 1A5 cells were thus biased to specifically express α2,6 Sia, which is not usually the case because α2,3 and α2,6 Sias are normally both expressed in tissues and cells at various different ratios [42]. We infected these cells with NA mutant viruses at low multiplicities of infection (MOI), and monitored their propagation kinetics by measuring FFU titers (S2 and S3 Figs).

**Fig 4:**
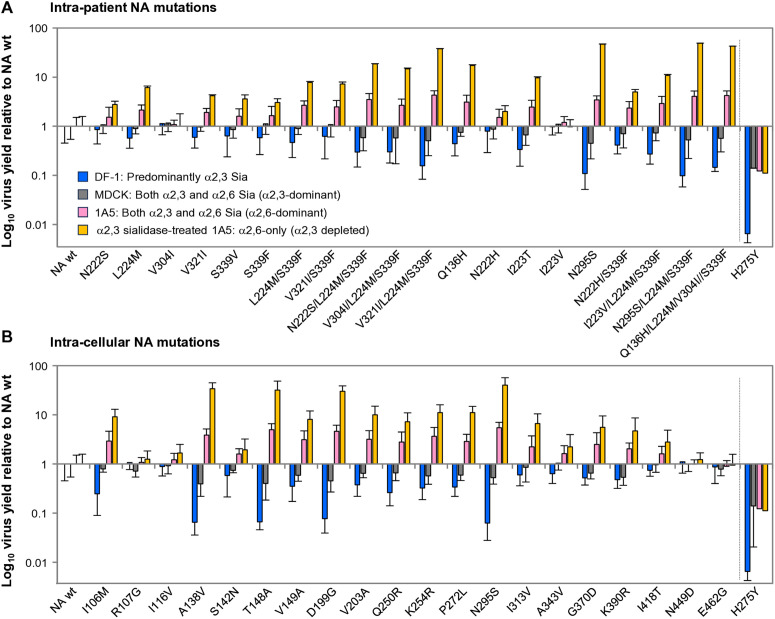
Sia-dependent replication of human-adaptive NA mutant viruses. Viral replication of (A) intra-patient NA mutants and (B) intra-cellular NA mutants was evaluated in four cell types with distinct α2,3/α2,6 Sia expression profiles. DF-1 cells predominantly express α2,3 Sia; MDCK cells express both α2,3 and α2,6 Sia with α2,3 dominance; 1A5 cells express both species with α2,6 dominance; and α2,3-sialidase-treated 1A5 cells selectively express α2,6 Sia, with α2,3 Sia depletion experimentally verified to persist for the full 13-h infection interval (1-h adsorption + 12-h incubation; S1 Fig). Cells were infected at low MOIs (MOI = 0.005 for DF-1 and MDCK; MOI = 0.05 for untreated or α2,3-sialidase-treated 1A5 cells). Virus yields in supernatants were quantified by FFU assay over 72 h (S2 and S3 Figs). The 12-h post-infection values—corresponding to the early replication phase within the validated α2,3-depleted window—are shown here as virus yields relative to NA-wt (= 1). Each data point represents the mean ± SD from three independent experiments. Because virus titers were measured exclusively from supernatants, these values reflect successful progeny virion release rather than total intracellular production.

Yields of NA mutant viruses at 12 hpi, which together with the 1-h adsorption period fall within the 13-h interval over which the α2,3-depleted state in sialidase-treated 1A5 cells was verified to persist, are shown in Fig 4. In DF-1 cells, many of the intra-patient-derived NA mutants had lower virus yield than the wt virus, and no mutants with increased yields were observed (Fig 4A). The same phenomenon was observed in MDCK cells, although the decreases in viral yields were more subtle. In contrast, in 1A5 cells, many of the intra-patient NA mutants exhibited up to 4-fold increased viral yields relative to wt virus. Notably, these effects were more prominent in α2,3-sialidase-treated 1A5 cells, with virus yields reaching more than 10-fold above those of wt virus. The H275Y reference virus exhibited markedly reduced virus yields in all cell types. The effects of the intra-cellular NA mutations on virus yields across all four cell types mirrored those of the intra-patient NA mutations (Fig 4B). These results demonstrate that adaptive NA mutations enhanced virus yields in human airway cells in an α2,6 Sia-dependent manner.

### Slightly but not strongly reduced sialidase activity is correlated with improved NA mutant virus yields

**Fig 5:**
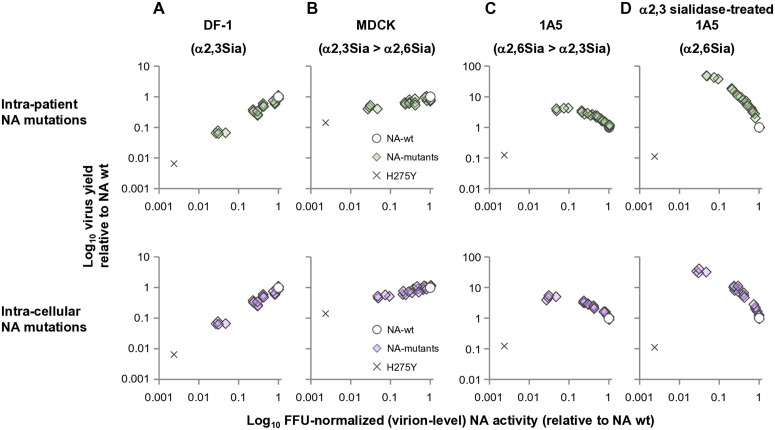
Relationship between FFU-normalized (virion-level, net) NA activity and Sia-dependent early viral replication of adaptive NA mutants. Scatter plots show the relationship between FFU-normalized (virion-level, net) NA activity (X-axis) and virus yields at 12 hpi (Y-axis) for NA mutant viruses measured in (A) DF-1 cells (α2,3-dominant), (B) MDCK cells (mixed, α2,3-dominant), (C) 1A5 cells (α2,6-dominant), and (D) α2,3-sialidase-treated 1A5 cells (α2,6-only; α2,3 depletion verified for the entire 13-h infection interval). Upper panels show intra-patient NA mutations; lower panels show intra-cellular NA mutations. NA-wt is plotted as an open circle at (1, 1). NA mutant viruses and the H275Y reference virus are plotted as colored diamonds and crosses, respectively. Values represent means from three independent experiments.

Because the N295S, N295S/L224M/S339F, A138V and D199G viruses had noticeably lower sialidase activities and also relatively higher virus yields in human cells, we evaluated the relationship between these two factors in four types of cells. In DF-1 and MDCK cells, yields of the intra-patient and the intra-cellular NA mutants were correlated positively with their sialidase activity (Fig 5A and 5B), and almost all NA mutants had reduced virus yields relative to the wt virus, depending quantitatively on the degree of reduction of sialidase activity. Data from the reference H275Y virus fitted the curve of the adaptive NA mutants. Conversely, in 1A5 cells, virus yields were negatively correlated with sialidase activity (Fig 5C); almost all NA mutants had enhanced virus yields depending on the degree of reduction of sialidase activity. This relationship was more pronounced in α2,3 sialidase-treated 1A5 cells (Fig 5D). Data on the H275Y virus plotted outside the fitted curve for the adaptive NA mutants. However, all plots in general, including H275Y virus, revealed that NA mutant viruses with sialidase activity reduced to within a specific range (1/10 of wt virus) had maximized virus yields. Taken together, these results suggest that the adaptive NA mutant viruses slightly reduced sialidase activity to within an optimal range, thereby enhancing viral replication in cells predominantly expressing α2,6 Sia.

### Distinct classes of adaptive NA mutations differentially impact protein expression and intrinsic catalytic function

To further explore the mechanisms by which mutant NAs reduced “net” NA activity of virus particles, we evaluated the effects of adaptive NA mutations on NA protein expression. We quantified mutant NAs in plasmid-transfected cells, because previous studies had shown that the amounts of mutant NAs in the clade 2.2 virus virions reflected intracellular expression levels [37]. Consistent with this, our analysis demonstrated strong correlations between intracellular NA expression and NA levels incorporated into purified virions (R^2^ = 0.7256 for intra-patient NA mutants and R^2^ = 0.7863 for inter-cellular NA mutants) (S4 Fig). As virion-based quantification exhibited substantially higher experimental variability, intracellular NA expression provided a more stable and reproducible surrogate for virion NA content. We therefore used NA expression levels in transfected cells as a surrogate measure of virion-associated NA levels to assess the effects of NA mutations on NA incorporation into virus particles.

**Fig 6:**
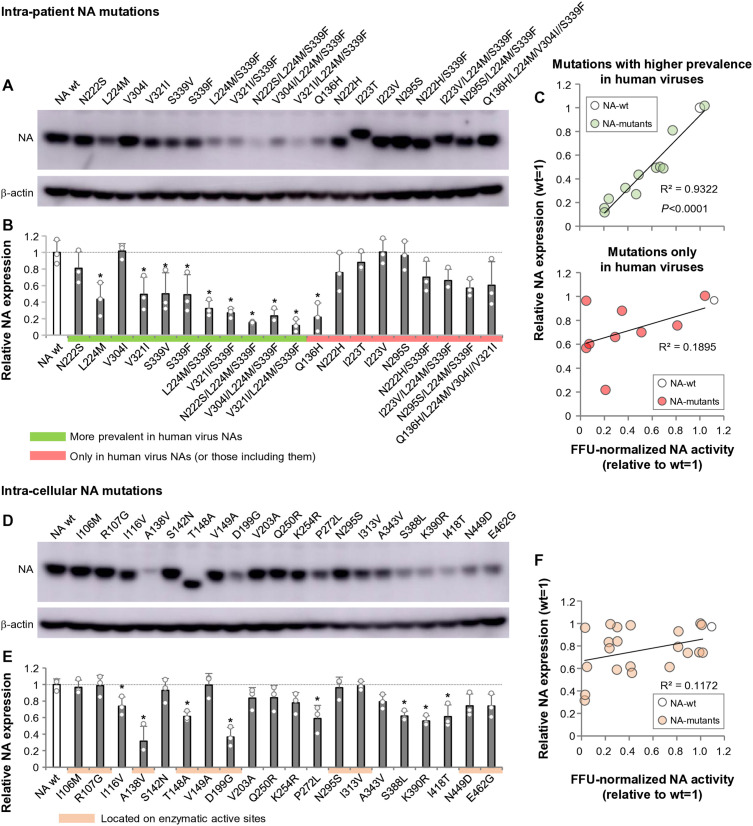
Effects of adaptive NA mutations on NA protein expression and their relationship to FFU-normalized (virion-level, net) NA activity. (A, B) Intracellular expression levels of intra-patient NA mutants and (D, E) intra-cellular NA mutants were quantified by Western blotting. At 24 h after transfection of Flag-tagged NA-expressing plasmids into 293T cells, lysates were collected and NA expression was analyzed. Band intensities of each NA mutant were normalized to β-actin in the same lane and expressed relative to NA-wt. (A, D) Representative Western blot images. (B, E) Quantified expression levels of NA mutants. Data represent mean ± SD from three independent experiments. *P < 0.01. (C, F) Relationship between FFU-normalized (virion-level, net) NA activity and intracellular NA expression for intra-patient (C) and intra-cellular (F) NA mutants. FFU-normalized NA activity values (X-axis) are plotted against intracellular NA expression levels (Y-axis). For intra-patient NA mutations (C), data are shown separately for two mutation categories: (C, upper) mutations detected more frequently in human-derived viruses than in avian-derived viruses, and (C, lower) mutations detected exclusively in viruses isolated from infected patients.

Western blotting showed that intra-patient mutant NAs were expressed in similar or decreased amounts relative to wt NA, and no increased expression was observed (Fig 6A and 6B). Scatter plots showed a strong correlation between FFU-normalized (virion-level, net) NA activity and protein levels in the group of mutations presumably transmitted from birds to humans (Fig 6C, upper panel). In contrast, for the mutations presumably selected during replication in infected patients, FFU-normalized NA activity did not correlate with the level of expression (Fig 6C, lower panel).

Similar to these intra-patient mutant NAs, the intra-cellular mutant NAs were expressed at unchanged or decreased levels relative to wt NA (Fig 6D and 6E). The amounts expressed also did not correlate with FFU-normalized NA activity (Fig 6F), mirroring the pattern observed for NA mutations selected in patients. These results suggest that decreased sialidase activity in NA mutant viruses can be attributed to reductions in NA protein abundance and/or intrinsic enzymatic activity.

To further distinguish these possibilities, we additionally quantified NA amount–normalized (intrinsic) sialidase activity by normalizing virion sialidase activity to the NA protein abundance measured from purified virions (S5 Fig). This analysis clarified that mutations with higher prevalence in human-derived viruses generally maintained intrinsic catalytic activity close to wt levels, indicating that their reduced virion-level activity primarily reflects decreased NA incorporation rather than impaired catalytic efficacy. In contrast, many mutations found exclusively in human-virus NAs or selected in human airway cells exhibited marked reductions in intrinsic catalytic activity, accompanied by modest decreases in NA protein abundance. Although both factors contribute to the overall reduction in virion-level sialidase activity, the magnitude of intrinsic activity loss was substantially greater than the change in protein amount, indicating that impaired enzymatic efficiency is the primary driver for these categories, with decreased NA abundance playing a secondary, reinforcing role. Collectively, these findings indicate that both reduced NA incorporation and reduced intrinsic activity shape NA adaptation, but that intrinsic catalytic impairment is disproportionately enriched among NA mutations selected in patients or human cells.

### Adaptive NA mutant viruses retain susceptibility to NAIs

**Fig 7:**
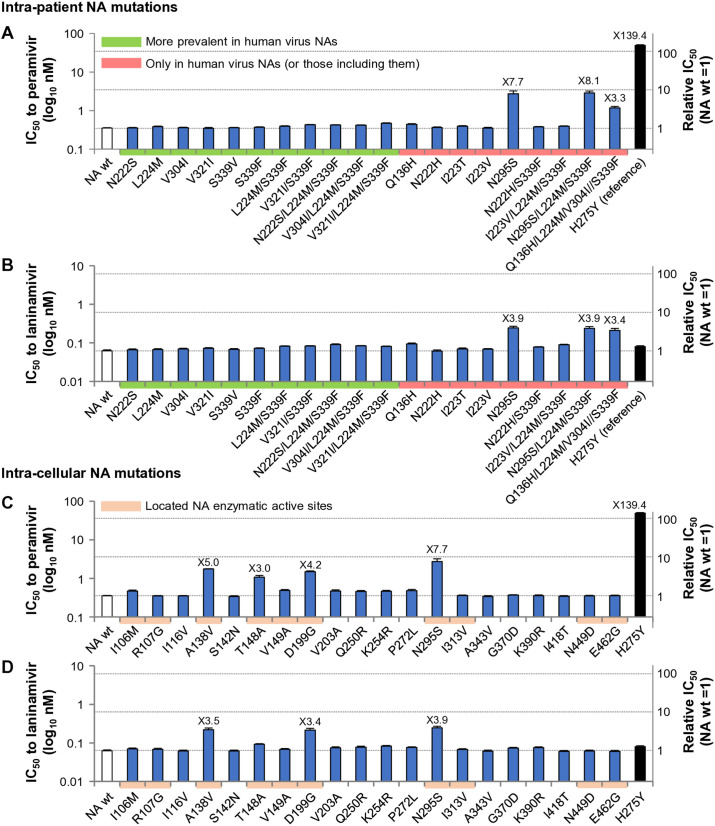
Effects of adaptive NA mutations on susceptibility to NA inhibitors. The effects of (A, B) intra-patient NA mutations and (C, D) intra-cellular NA mutations on the susceptibility of clade 2.2 viruses to peramivir (A, C) and laninamivir (B, D) were measured by chemiluminescent NA inhibition assay using the NA-XTD substrate. Serial dilutions of each virus preparation were first analyzed to determine NA activity, and the dilution yielding a signal-to-noise ratio of 40 was selected as the standardized activity-normalized input for inhibitor titrations. The left Y-axis shows absolute IC₅₀ values (log_10_ nM), and the right Y-axis shows values relative to NA-wt. H275Y, a major mutation conferring resistance to peramivir was included as a reference control. Data represent mean ± SD from three independent experiments.

Virus susceptibility to NAIs and sialidase activity are often altered concomitantly by introduction of NA mutations [28]. We therefore evaluated the susceptibility of NA mutant viruses to peramivir and laninamivir by a chemiluminescence NA inhibition assay using NA-XTD as the substrate. In accordance with the NA-XTD protocol and international NAI testing guidelines, virus input for each mutant was standardized by NA activity rather than by FFU; specifically, the dilution yielding a signal-to-noise ratio of 40 in the NA-XTD assay was used for IC_50_ determination (see Methods). We also included the peramivir-resistant virus with mutation H275Y as a reference [43,44]. The IC_50_ values of wt virus for peramivir and laninamivir were 0.35 nM and 0.062 nM, respectively (Fig 7), i.e., within the IC_50_ range of many H5N1 viruses in the literature [44]. This confirmed that the wt virus was a typical H5N1 virus with high susceptibility to both NAIs.

Among the intra-patient NA mutations, N295S and N295S/L224M/S339F moderately increased the IC_50_ for peramivir by 7.7- and 8.1-fold, respectively, and for laninamivir by 3.9-fold, but all the mutant viruses remained highly susceptible to both drugs (Fig 7A and 7B). Among intra-cellular NA mutations, A138V and D199G increased IC_50_ for peramivir by 5- and 4.2-fold, respectively, and for laninamivir by 3.5- and 3.4-fold, respectively, but here also all the mutant viruses remained highly susceptible to both drugs. These results suggest that the adaptive NA mutations did not lead to marked phenotypic changes regarding NAIs and the viruses retained inherent drug-susceptibility.

### Adaptive NA mutant viruses exhibit increased binding to α2,6 Sia

**Fig 8:**
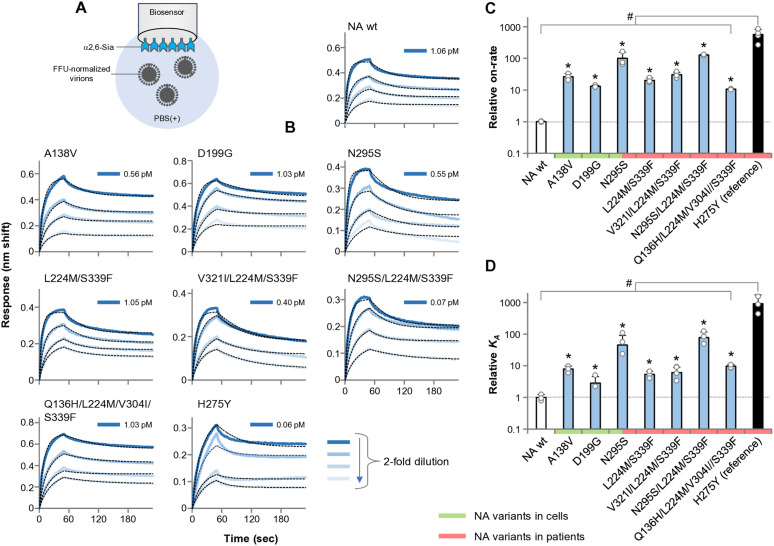
Binding characteristics of adaptive NA mutant viruses to α2,6 Sia measured by BLI. (A) Schematic overview of the BLI assay. Virus preparations were applied to sensors coated with α2,6 Sia. Because BLI measurements were performed in PBS (+) containing cations required for NA activity, the apparent association constant (*K*_*A*_) and on-rate (*K*_*on*_) reflect the combined effects of HA-mediated binding and NA-mediated receptor dissociation of virions. (B) Representative sensorgrams showing binding of FFU-normalized NA mutant viruses to α2,6 Sia. The numeric value shown beside the dark blue line in each panel indicates the highest apparent virion concentration used as the starting point of the two-fold dilution series for that sample (calculated from FFU-based infectious particle equivalents). Dotted lines indicate fitted median values derived from nonlinear regression analysis and are shown to visualize the estimated parameter used for statistical comparisons. (C) Relative on-rate values of NA mutant viruses. (D) Relative *K*_*A*_ values of NA mutant viruses. Data represent mean ± SD from three independent experiments. *P < 0.01. # indicates that the H275Y mutant exhibited values significantly different from all other NA mutants as well as NA-wt (*P* < 0.01). Statistical analysis was performed using one-way ANOVA with Tukey’s multiple comparison test.

The balance between the opposing functions of HA-binding and NA-dissociation is important for influenza virus attachment to host cells [14,28]. We thus evaluated the effect of decreased sialidase activity of the adaptive NA mutants on virus binding to α2,6 Sia. To this end, we performed biolayer interferometry (BLI) using α2,6 Sia and purified mutant viruses as the ligand and analyte, respectively (Fig 8A). Because the assays were conducted in PBS (+) containing cations necessary for NA activity, we analyzed the binding profiles of virus particles as the sum of HA-binding and NA-dissociation. Based on the phenotypic effects of the adaptive NA mutations (Figs 3–5), 5 intra-patient NA mutants (N295S, L224M/S339F, V3221/L224M/S339F, N295S/L224M/S339F, Q136H/L224M/V304I/S339F) and 3 intracellular NA mutant viruses (A138V, D199G, N295S) were selected for further study (N295S was present in both groups, resulting in total of 7 mutants). H275Y was also included as a reference.

Typical sensorgrams of NA mutant viruses are shown in Fig 8B. The on-rate of the NA mutant viruses increased by nearly two orders of magnitude compared to the wt virus, but was still less than one-fifth of the H275Y reference virus (Fig 8C). The association constant (*K*_*A*_=on-rate/off-rate) of the NA mutant viruses reflected the change in on-rate (Fig 8D). These results indicate that the NA mutant virus had reduced sialidase activity to compensate for low α2,6 Sia binding activity, thereby conferring an increase in Sia binding of virus particles.

### Co-occurring HA and NA adaptive mutations cooperatively promote α2,6 Sia-mediated viral fitness

**Fig 9:**
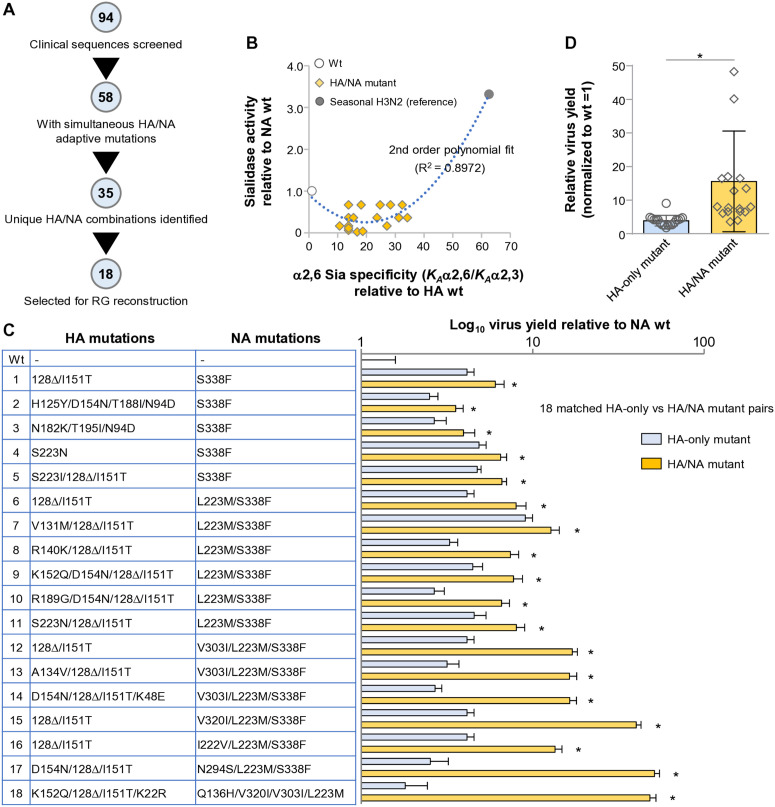
Cooperative effects of HA- and NA-adaptive mutations on α2,6 Sia-dependent viral replication. (A) Screening of Egyptian clade 2.2.1 H5N1 clinical sequences for co-occurring HA- and NA-adaptive mutations. Among 94 patient-derived sequences, 58 isolates encoded at least one HA-adaptive mutation reported in previous study [18] together with one or more NA-adaptive mutations identified in this study. From these, 35 unique HA/NA mutation combinations were identified, and 18 representative HA/NA pairs were selected for reconstruction by reverse genetics. (B) Relationship between HA α2,6 Sia binding preference and FFU-normalized (virion-level, net) NA activity among the 18 reconstructed HA/NA simultaneous mutants. HA α2,6-binding preference was determined from previously reported solid-phase direct binding data [18], using the ratio *K*_*A*_(α2,6SLN2)/*K*_*A*_(α2,3SLN1) as an index of α2,6 specificity. As an external reference for a fully human-adapted phenotype, the seasonal H3N2 virus A/Japan/434/2003 was included for comparison. NA activity was measured by chemiluminescent NA assay and normalized to NA-wt. A centered second-order polynomial model accounted for 89.7% of the variance (R² = 0.8972). Although NA activity generally increased with greater HA α2,6-binding preference, HA/NA combinations within the limited α2,6-binding range characteristic of early human-adaptation variants exhibited locally reduced NA activity. (C) Comparison of viral yields of HA-only and HA/NA simultaneous mutants in α2,3-sialidase-treated 1A5 cells (α2,6-only). Cells were infected at an MOI of 0.05, and virus yields were measured at 12 hpi—the early replication phase corresponding to 13 h after sialidase treatment, during which α2,3 Sia depletion was experimentally validated to persist. Viral yields are shown relative to wt (= 1). Across all 18 mutation pairs, HA/NA simultaneous mutants exhibited significantly higher viral yields than their matched HA-only mutants, demonstrating cooperative enhancement of α2,6 Sia–dependent replication. (D) Summary comparison of HA-only versus HA/NA simultaneous mutants. Aggregate analysis confirmed significantly increased replication of HA/NA simultaneous mutants under α2,6-dominant conditions. *P < 0.01.

Because HA and NA act in a coordinated manner during viral entry, we next examined whether the adaptive NA mutations identified in this study cooperate with HA mutations previously shown to enhance α2,6 Sia binding. In our earlier work on Egyptian clade 2.2.1 H5N1 viruses isolated from infected patients [18], multiple HA mutations conferring increased α2,6 Sia preference were identified. We therefore re-analyzed all available Egyptian patient-derived H5N1 sequences to determine whether these HA-adaptive mutations co-occur with the NA-adaptive mutations characterized in the present study. This survey revealed that 56 of 94 clinical virus isolates encoded at least one HA-adaptive and one NA-adaptive mutation simultaneously (Fig 9A and S1 Table), and 35 distinct HA/NA mutation combinations were detected (S2 Table).

To experimentally assess the functional consequences of such simultaneous mutations, we selected 18 representative HA/NA combinations in which each HA mutation increased α2,6 Sia binding and the corresponding NA mutation reduced sialidase activity. Using reverse genetics, we generated recombinant viruses carrying each HA/NA double mutation, as well as the corresponding 18 HA-only mutant viruses for direct comparison. We first analyzed the relationship between altered HA α2,6 Sia binding preference and NA sialidase activity among these 18 HA/NA mutants (Fig 9B). Although all HA mutations modestly increased α2,6 Sia specificity relative to the wt H5N1, their α2,6 binding remained substantially lower than that of a representative seasonal H3N2 strain (A/Japan/434/2003). Conversely, the NA-adaptive mutations conferred a mild but significant reduction in sialidase activity, contrasting sharply with the high NA activity of the H3N2 reference virus. A centered second-order polynomial model explained 89.7% of the variance (R^2^ = 0.8972), with a significant positive quadratic term, indicating that NA activity generally increases in an upward-curving manner as HA α2,6-binding specificity becomes stronger. Notably, within the limited α2,6-binding range characteristic of the simultaneous HA/NA mutants detected in patients, several variants occupied a locally concave region in which NA activity was moderately decreased. These findings suggest that the HA/NA mutation pairs detected in patients represent very early-stage human-adaptation intermediates, positioned in a transitional zone before acquisition of the high α2,6-binding and high NA-activity profile typical of seasonal human influenza viruses.

Finally, we compared the viral yields of the 18 HA-only and 18 HA/NA simultaneous mutants in α2,3-sialidase-treated 1A5 cells under conditions where α2,3 Sia depletion persisted for the entire 13-h assay window, as in Fig 4. In this α2,6-dominant context, HA/NA double mutants consistently exhibited significantly greater virus yields than their HA-only counterparts (Fig 9C). Statistical comparison of the two groups confirmed that NA mutations acted cooperatively with HA mutations to further enhance α2,6 Sia-dependent viral growth (Fig 9D). Together, these results indicate that, among the variants actually selected in infected patients, adaptive NA mutations augment the replication advantage conferred by α2,6 Sia-directed HA mutations, supporting a coordinated HA–NA adaptation process during the early stages of human infection.

### Adaptive mutations that affect sialidase activity and their marginal energetic effects are located near the tetramer interface or active site

**Fig 10:**
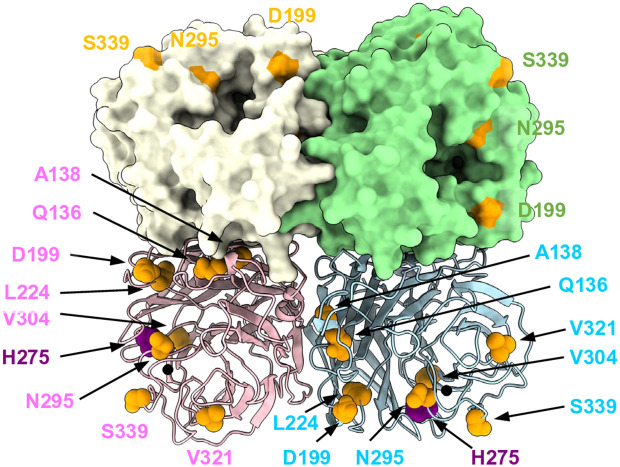
Mutation sites in the structure of the NA tetramer. The NA tetramer is shown as a cartoon model (Chains A and C) and surface models (Chain B and D). Mutation sites are depicted as orange spheres, whereas the reference mutation site (H275) is shown in purple. Calcium ions are depicted as black spheres.

To better understand the context of these NA mutations within the protein structure, we mapped the identified adaptive mutations onto an NA structure (Fig 10). Among the seven sets of mutations that we characterized experimentally (Fig 8), six sets were located at the interfaces of the tetramer, and one mutation (N295S) was located near the active site. Notably, A138V, D199G, and N295S exhibited the most pronounced loss of sialidase activity (Fig 3). This is likely due to the specific locations of these mutations: A138 is well-buried (relative solvent accessible surface area = 0) and situated at the center of the tetramer. Although the energetic effect of the A138V mutation is nearly marginal (computed ΔΔG = 0.6), mutating the Ala to Val might affect the equilibrium between the open and closed conformations of the tetramer. D199 forms a salt bridge with K150; therefore, mutating D199G would result in the loss of this interaction, leading to destabilization of the tetramer (computed ΔΔG = 40.3). N295 is located near the active site. Although the energetic effect of N295S on the stability of the NA tetramer itself was also marginal (computed ΔΔG = 3.8), a mutation in this region would directly affect sialidase activity. Interestingly, the mutation that caused the greatest deterioration, H275Y, a reference mutation in our study, also had a marginal energetic effect on the stability of the NA tetramer (computed ΔΔG = 1.0). Together with the experimental characterization mentioned above, these results imply a complex interplay between the catalytic activity, stability, and binding capability of the NA tetramer. These properties appear to counterbalance each other upon the introduction of adaptive mutations.

## Discussion

To date, the H5N1 virus has acquired multiple human-adaptive mutations in several genes during its infection in the field or in infected patients [18,20,34,45,46]. Many studies have searched for adaptive mutations in HA and polymerase [19,21,33,34], whereas studies on NA have intensively focused on NAI escape mutants [35,36], with a few exceptions investigating adaptive NA mutations [37,47]. In this context, to the best of our knowledge, the present report is the first to systematically characterize adaptive NA mutants selected for in H5N1 virus-infected patients, by comparing their phenotypic traits with adaptive NA mutants selected for in human cells in vitro.

In this study, no NAIs were added to the culture medium during cell growth and clade 2.2 diversification (Fig 1). Nevertheless, the observed phenotypes of intra-cellular-derived NA mutants were all consistent with NA mutants isolated from patients (Figs 3–8). This implies that the identified intra-patient NA mutations included no, or at least negligible, escape mutations with the effects indistinguishable from adaptive mutations. In fact, among the single NA mutations identified in patients (Q136H, N222H, I223T, I223V, N295S), only N295S minimally altered IC_50_ values to NAIs. N295S was already known to moderately reduce the susceptibility of H5N1 and seasonal H1N1 and H3N2 viruses to NAIs [36]. Intriguingly, however, in clade 2.2-infected patients, N295S was detected in NA of viruses from clinical swabs before NAI administration [48]. This implies that the N295S mutation occurred in the infected individuals without escape-selective pressure from NAIs. These findings further suggest that the intra-patient NA mutations we identified here arose as a result of selective pressure specific for adaptation to the human host. This is consistent with our null hypothesis, and also provides an answer to the question why the N295S mutation occurred in clade 2.2-infected patients before NAI administration. Nevertheless, because most medical records of the therapies received by the clade 2.2-infected patients, such as detailed information on NAI administration, were with a few exceptions not available to us [49], we cannot completely exclude the possibility that the intra-patient NA mutations included potential NAI escape mutations. To completely exclude this possibility, further analyses will be required that integrate clinical data of each infected patient and relevant viral gene sequences.

The intra-patient and intra-cellular NA mutants were shown to moderately decrease FFU-normalized (virion-level, net) NA activity to within a narrow range (about 1/10 of the wt virus) (Fig 3), which was advantageous for virus yields in human cells (Fig 5) and also virus binding profiles to α2,6 Sia (Fig 8). This is in line with an in vitro study [47] showing that H5N1 viruses acquired adaptive NA-E119V mutations to moderately reduce sialidase activity during serial passage in primary human cells. Conversely, when sialidase activity was further reduced by >10-fold by the H275Y mutation, virus yield was greatly reduced relative to NA-wt, consistent with other reports showing H5N1 virus attenuation by this mutation [50]. Also, in the present study, there appeared to be an optimal range of reduced sialidase activity that resulted in maximal virus titers in human cells (Fig 5). Taken together, these findings suggest that the adaptive NA mutants optimally reduced NA sialidase activity in accordance with the weaker HA binding to α2,6 Sia during viral infection of human cells — a kind of “fine-tuning”.

Our Western blotting analyses, together with FFU-normalized (virion-level, net) NA activity assays and NA amount–normalized (intrinsic) NA activity measurements, revealed two mechanisms responsible for the reduced sialidase activity of adaptive NA mutants. The first is decreased protein expression due to NA mutations (Fig 6). Given the strong correlation between sialidase activity and protein expression, it is likely that group of mutations that facilitated transmission from birds to humans predominantly though this mechanism. This aligned with a previous report indicating that the human-like clade 2.2 NAs carried L204M mutation, which reduced protein expression on the virion, thereby lowering NA activity [37]. The second mechanism is a functional decline in sialidase activity itself due to NA mutations (S5 Fig). This was observed sporadically in several NA mutations selected for in patients and human cells, which may be attributed to more direct selective pressure for human-adaptation in these environments. In summary, these findings suggest that mutant NA viruses in patients have decreased virion sialidase activity both quantitatively and qualitatively in H5N1 virus-infected patients.

The functional HA–NA balance is crucial for influenza virus attachment to host cells [14,27–32]. Indeed, biolayer interferometry demonstrated that EG/D1, which possesses an avian-like HA with high α2,3 Sia affinity but only low α2,6 Sia affinity [20], increased the overall α2,6 Sia binding affinity of virus particles by reducing sialidase activity (Fig 8). In the same manner, when typical avian influenza viruses infect the human upper airway, they are inefficiently taken up, likely because their original NA activity is stronger than the weakened Sia binding activity in this environment. These findings suggest that H5N1 viruses acquire adaptive NA mutations that reduce virus-dissociating activity to counterbalance the weaker Sia binding ability in humans, thereby enhancing α2.6 Sia-tropism of virus particles. This notion aligns with a previous hypothesis regarding H5N1 virus adaptation to humans [47]. Also, because all replication measurements in this study were obtained from infectious virions released into the culture supernatant, these data inherently reflect successful progeny virion dissociation from sialylated cell surfaces. Thus, the comparable or enhanced titers observed for most adaptive NA mutants indicate that partial reductions in NA activity do not impair virion release under the cellular conditions tested.

In addition to these NA-driven adjustments in HA–NA balance, our analysis of clinical H5N1 isolates revealed that adaptive NA mutations frequently co-occur with HA mutations that confer only modest increases in α2,6 Sia binding. Among 94 Egyptian patient-derived viruses, 56 encoded at least one HA- and one NA-adaptive mutation simultaneously, forming 35 distinct HA/NA combinations. Functional evaluation of 18 naturally occurring HA/NA mutation pairs demonstrated that these variants occupied a narrow early-adaptation window, defined by weak but detectable α2,6 binding and locally reduced NA activity—levels far below those characteristic of human seasonal viruses. Under experimentally α2,6-dominant conditions in human airway-derived cells, viruses bearing both HA and NA mutations exhibited improved replication compared with their corresponding HA-only counterparts, whereas the parental HA background required NA adaptation to display measurable effects. Importantly, this cooperative phenotype was restricted to the weak-binding range representative of early adaptation intermediates and did not reflect the affinity profiles associated with fully human-adapted viral phenotypes.

Because α2,3- and α2,6-Sias are co-expressed in most human airway tissues at varying ratios [42], it is noteworthy that the most pronounced replication increases (>10-fold) were observed only under experimentally α2,3-depleted conditions (Figs 4 and 5). In contrast, replication advantages under physiologically mixed receptor environments were modest. Thus, in natural infection settings, the phenotypic contribution of NA adaptation is likely incremental and less substantial than that of previously characterized HA or polymerase adaptations reported in ferret transmission studies [17,51–53] or naturally occurring human isolates [18,33]. Nevertheless, the presence of naturally co-occurring HA–NA mutation pairs supports a model in which NA contributes to early-stage tuning of HA–receptor interactions during the initial steps of host-range expansion.

For avian influenza viruses to achieve pandemic potential, a shift in receptor specificity from α2,3- to α2,6-Sia is required [16]. Numerous HA mutations affecting this property have been identified in ferret adaptation experiments [51,52] and patient-derived H5N1 strains [18]. But, importantly, most of these mutant HA viruses had only very low α2,6 Sia binding avidity relative to seasonal viruses [22–25]. This implies that avian influenza viruses undergo a transitional period, in which they infect humans with suboptimal avidity before gradually switching their receptor tropism, as seen in the 1968 pandemic caused by the H3N2 virus [26]. In fact, unlike bird-derived H5N1 viruses, human-derived H5N1 viruses often have lower sialidase activity (due to NA stalk deletion) [54,55], whereas seasonal influenza viruses have full-length NAs with strong sialidase activity. Our findings indicate that the complementary NA mutation mechanism may contribute only transiently during this early transition, supporting receptor–context-dependent replication before high-affinity HA adaptation is fully established.

Because this study investigates amino acid substitutions that modestly enhance H5N1 replication in human-like cellular environments, we carefully assessed its relevance to Dual-Use Research of Concern (DURC). All experiments were performed using recombinant EG/D1 viruses containing the avian-tropic clade 2.2 HA, which shows minimal α2,6 Sia engagement and does not support efficient infection or transmission in mammals. Notably, the phenotypic effects of NA mutations were detectable only under experimental condition with near-complete depletion of α2,3 Sia—a scenario not found in natural human tissues. In vivo, where α2,3- and α2,6-Sia coexist, their impact remained limited and substantially weaker than that of well-characterized human-adaptive HA substitutions (e.g., Q226L or G228S). While some NA mutations exhibited effects comparable to early weak HA adaptations identified in clade 2.2 patient isolates, multiple functional observations suggest that these effects remain auxiliary and do not provide evidence of enhanced transmissibility or pathogenicity. All work was performed under biosecurity level 3 (BSL3) containment and reviewed by our institutional biosafety and DURC oversight committees, which concluded that the study does not meet DURC criteria. We include this disclosure to ensure transparency and contextual framing of the public-health relevance of these findings.

Collectively, our results indicate that NA mutations identified in clade 2.2 human H5N1 infections do not independently confer human adaptation but instead act as incremental modifiers whose functional impact becomes apparent primarily when paired with HA variants exhibiting weak α2,6 specificity. By integrating evolutionary analysis, quantitative phenotyping, and receptor–context-dependent infection models, this study provides mechanistic insight into how early-stage HA–NA co-adaptation may proceed during the initial bottlenecks encountered when avian influenza viruses infect humans. These findings highlight the value of monitoring coordinated HA–NA evolutionary trajectories rather than evaluating each gene in isolation when assessing zoonotic influenza virus risk. Continued genomic surveillance and functional risk assessment incorporating HA–NA pairing will be essential to improve pandemic preparedness frameworks.

## Materials and methods

### Ethics statement

All experiments using live H5N1 viruses were conducted at BSL3 at Kyoto Prefectural University of Medicine. All studies using recombinant H5N1 viruses were conducted in accordance with relevant laws in Japan and approved by the Biological Safety Committee of Kyoto Prefectural University of Medicine (approval number 30–104), after a risk assessment by the Living Modified Organisms Committee of Kyoto Prefectural University of Medicine, and, if necessary, the Ministry of Education, Culture, Sports, Science, and Technology of Japan.

### Cells

MDCK cells and chicken embryo fibroblast (DF-1) cells were obtained from the American Type Culture Collection. 293T cells were obtained from RIKEN BioResource Center Cell Bank. We previously established 1A5 cells from primary human airway cells, in which the predominant expression of α2,6 Sia over α2,3 Sia was confirmed [39]. CEFs were prepared from embryonated eggs obtained from Shimizu Laboratory Supplies, Japan, as previously reported [19]. They were maintained in Dulbecco’s modified Eagle’s medium (DMEM) with 10% fetal calf serum. Primary human small airway epithelial cells (Lonza Corporation) were cultured under a non-air-liquid interface, according to the manufacturer’s recommendation.

### Biosecurity and biosafety

All personnel conducting this study were vaccinated against H5N1 influenza virus. In this study, the infection investigations were conducted as a single infection as is routinely done in many other studies [20,33,56]. No passage or transmission experiments were performed in this study.

### Viruses and reverse genetics

Recombinant H5N1 viruses were generated by reverse genetics based on the genetic background of EG/D1, a representative clade 2.2 virus whose HA displays typical avian-type receptor binding (high α2,3 Sia affinity and only weak α2,6 Sia affinity) [20]. Mutant viruses were generated by site-directed mutagenesis, and rescued in 293T/MDCK-SIAT1 co-cultures. To avoid passaging-associated selection, rescued viruses were propagated only once in MDCK-SIAT1 cells, purified by ultracentrifugation, and titrated by focus-forming assay [33] on MDCK-SIAT1 cells.

For analyses of NA-specific adaptation (Figs 3–8), NA mutations were selected as described below. For experiments assessing cooperative HA–NA adaptation (Fig 9), previously characterized HA mutations that modestly increase α2,6 Sia binding in Egyptian clade 2.2.1 H5N1 viruses [18] were incorporated into the EG/D1 HA backbone. Database screening of Egyptian clade 2.2.1 human isolates identified 35 HA/NA mutation combinations (S1 and S2 Tables), from which 18 representative pairs were selected based on (i) reported HA receptor-binding effects [18], and (ii) NA sialidase phenotypes in this study. For each selected HA/NA combination, two recombinant viruses were generated: (1) an HA-only mutant virus, and (2) an HA/NA simultaneous mutant virus.

The complete coding regions of both HA and NA in all recombinant viruses—including NA-only, HA-only, and HA/NA simultaneous mutants—were verified by Sanger sequencing prior to use. Because all recombinant viruses were derived from identical plasmid backbones and were not passaged beyond initial rescue, the six internal gene segments (PB2, PB1, PA, NP, M, and NS) remained genetically identical across all viruses. Therefore, all observed phenotypic differences can be attributed solely to the intentionally introduced HA and/or NA mutations.

### Identification of adaptive NA mutations in clade 2.2-infected patients

We sought NA mutations presumably selected in H5N1 virus-infected patients by database searching of NA sequences in human and bird clade 2.2 viruses in Egypt during 2006–2012. This yielded 94 human-derived NA sequences and 343 bird-derived NA sequences from the GISAID database (https://gisaid.org). NA mutations in the 94 human and 343 bird clade 2.2 strain were identified by comparing each NA sequence with the consensus sequence determined by aligning all the NA sequences. The criteria for selecting adaptive NA mutations were as follows. First, we selected NA mutations that were not detected in the bird-derived sequences, but were detected in human-derived sequences. This set of mutations was likely to have emerged within infected individuals. Second, we selected NA mutations that were detected more frequently in bird sequences than human sequences. Viruses with any of this set of mutations presumably emerged in birds and were subsequently transmitted to humans with high efficiency.

### Identification of adaptive NA mutations in primary human cells

HAE cells and CEFs as a control were infected with the recombinant EG/D1 virus at MOIs of 0.1 and 0.01, respectively. We employed low-MOI infection (0.01–0.1)—a widely used condition for studying influenza diversification and host adaptation in human airway epithelial cells—to enable reliable detection of de novo mutations arising during a near–single-round infection, while minimizing multiplicity re-infection that could obscure selective pressures acting on newly generated variants [57–60]. Before the experiment, the gene of the recombinant EG/D1 sample was sequenced in-depth by Sanger sequencing of 300 randomly selected clones obtained by RT-PCR. There were no clones with NA mutations, confirming sufficiently high gene homogeneity of the virus sample (the potential mutation rate was < 0.3%). After 96 hpi, genetic heterogeneity of progeny virus quasispecies in the supernatant was analyzed by randomly selecting 30 clones obtained by RT-PCR and performing Sanger sequencing (mutation detection threshold was 3%).

### Sialidase assay

We performed a chemiluminescent sialidase assay using NA-XTD as the substrate (NA-XTD Influenza Neuraminidase assay Kit, Applied Biosystems) and NA mutant viruses that were normalized by FFU titers, as previously described [61]. We adopted this assay because its high sensitivity was advantageous for detecting potentially low sialidase activity of EG/D1 viruses with an NA stalk deletion [20]. To additionally quantify “NA amount–normalized (intrinsic) NA activity,” equivalent volumes of FFU-normalized virion samples were further normalized by NA protein abundance determined by Western blotting, and sialidase activity was expressed relative to NA quantity (i.e., activity per unit NA protein). This complementary analysis enables assessment of intrinsic catalytic efficiency of mutant NAs independent of differences in NA incorporation levels into virions.

### Fluorescent lectin staining and α2,3-sialidase treatment

To characterize the distribution and temporal stability of α2,3- and α2,6- Sias, DF-1, MDCK, untreated 1A5, and α2,3-sialidase-treated 1A5 cells were subjected to fluorescent lectin staining. For α2,3-sialidase treatment, 1A5 cells were incubated with α2,3-specific sialidase (Vector Laboratories) under previously established conditions [62] (1 h, 37°C), washed three times with PBS, and returned to culture medium. For the time-course analysis, 1A5 cells were fixed immediately (0 h) or 13 h after sialidase treatment to assess the persistence of α2,3 Sia depletion.

Cells were seeded into 96-well tissue culture plates, washed with PBS, and fixed with 4% paraformaldehyde for 15 min at room temperature. After three washes, cells were incubated with fluorescein-conjugated MAL-I (Vector Laboratories), which recognizes α2,3 Sia, and Cy3-conjugated SNA (Vector Laboratories), which recognizes α2,6 Sia. Lectins were diluted in PBS containing bovine serum albumin according to the manufacturers’ instructions. Following lectin incubation, cells were washed with PBS and counterstained with Hoechst 33342 (ThermoFisher).

Fluorescence images for MAL-I, SNA, and Hoechst channels, along with merged images, were acquired using an FV3000 confocal microscope (Olympus) under identical exposure settings.

### Virus growth kinetics in cultured cells

MDCK cells and DF-1 cells were infected with wt or mutant NA virus at an MOI = 0.005. Untreated or α2,3-sialidase-treated 1A5 cells were infected at an MOI of 0.05. After 1-h virus adsorption, inocula were replaced with EMDM/F12 medium (ThermoFisher). Supernatants were collected over time for up to 72 h, and virus titers were determined by the FFU assay described above.

For comparative analyses of HA-only and HA/NA simultaneous mutants (Fig 10C), viral yields were quantified at 12 h post-infection (corresponding to 13 h after sialidase treatment), a time window during which α2,3 Sia depletion in 1A5 cells was confirmed to persist.

### Quantification of NA levels in transfected cells

Flag-tagged NA-wt and mutant plasmids were constructed in the pcXN2 backbone and transfected into 293T cells seeded in 24-well plates. Cells were harvested 24 h post-transfection and lysed in lysis buffer. Lysates were subjected to SDS-PAGE and Western blotting using anti-Flag antibody (Sigma-Aldrich) and Alexa Fluor 488-conjugated secondary antibody (ThermoFisher). β-actin was detected as a loading control. NA band intensities were normalized to β-actin and expressed relative to wt NA.

### Quantification of NA levels in purified virions

To quantify NA protein incorporated into virions, clarified culture supernatants were layered onto a 20% sucrose cushion and centrifuged at 28,000 × g for 2 h at 4°C. Pelleted virions were resuspended in PBS, and the preparations were normalized by FFU to ensure equivalent infectious units across samples. Equal FFU amounts of virions were mixed with SDS sample buffer, heated at 95°C for 5 min, and subjected to SDS-PAGE and Western blotting. NA was detected using an anti-NA antibody (Sino Biological), and NP was detected as a loading control. NA intensities were normalized to NP in the same lane and expressed relative to wt virus. Virion-associated NA levels were compared with intracellular NA expression levels obtained as described above.

### Western blotting and band quantification

For both transfected-cell and virion samples, bands were visualized using Amersham ECL Select reagent and imaged with an Amersham Imager 680 (Cytiva). Band intensities were quantified using Amersham Imager 680 software. All normalization procedures are described in the respective sections above.

### NAI susceptibility assay

Virus susceptibility to the NAIs peramivir (Peramivir Trihydrate, Selleck) and laminamivir (MedChemExpress), was determined using Chemiluminescent NA inhibition assays (NA-XTD Influenza Neuraminidase Assay Kit, Applied Biosystem), as described elsewhere [63]. In accordance with internationally accepted principles recommended by World Health Organization (WHO)—specifically, that inhibitor titrations should be performed using an activity-normalized virus input rather than a particle-normalized input—we followed the quantitative protocol of the National Institute of Infectious Diseases (NIID, Japan), which operationalizes this requirement. Serial dilutions of each virus preparation were first assayed to determine NA activity. The dilution yielding a signal-to-noise ratio of 40, ensuring sufficient enzyme activity and linear reaction kinetics, was identified for each sample. This activity-normalized dilution was then used as the standardized virus input for all subsequent inhibitor titrations. This procedure ensures that IC₅₀ values reflect the intrinsic inhibitor susceptibility of neuraminidase, independent of differences in FFU titers, particle infectivity, or NA incorporation levels among mutants. To evaluate virus susceptibility to NAIs, we used WHO criteria based on fold-change; for Influenza A viruses, an increase in IC_50_ value of <10-fold is defined as normal susceptibility, 10–100-fold as decreased susceptibility, and >100-fold as highly reduced susceptibility.

### BLI analysis

Profiles of the binding between NA mutant viruses and α2,6 Sia were assessed by biolayer interferometry using the BLITz System (Sartorius/ForteBio). α2,6 Sia (α2,6 sialylglycopeptide, FUSHIMI Pharmaceutical Co Ltd) was biotin-labeled using EZ-Link Sulfo-NHS-LC-LC-Biotin (ThermoFisher), purified with Zeba Spin Desalting Columns 7K MWCO (ThermoFisher), and then dialyzed against PBS (+). Biotin labeling efficiency was calculated using the Fluorescence Biotin Quantitation Kit (ThermoFisher) to confirm that the labeling ratio was an ideal 1:1. The biotinylated α2,6 Sia was immobilized on Octet Streptavidin (SA) Biosensor (Sartorius/ForteBio) in a saturated state and used as a ligand. Apparent mole numbers of NA mutant viruses were calculated from FFU titers. The highest concentration shown in Fig 8 (e.g., “1.06 pM” for NA-wt) represents the calculated apparent virion concentration used as the starting point for a two-fold serial dilution series. The viruses and α2,6Sia were reacted in PBS (+) buffer, where HA binding and NA dissociating were assessed. Thus, virus binding to α2,6 Sia was measured as the sum of the HA binding activity and NA dissociating activity of virus particles. Apparent association constants (*K*_*A*_) and on-rates were measured by global-fitting of the data.

### Assessment of HA α2,6 Sia binding preference

The α2,6 Sia binding properties of the HA mutants used in this study were obtained from our previous report [18] under CC-BY licensing. As described therein, receptor binding specificity was measured using a solid-phase direct binding assay with defined sialylglycopolymers. Binding curves were fitted to determine apparent *K*_*A*_, and the ratio *K*_*A*_(α2,6SLN2)/ *K*_*A*_(α2,3SLN1) was used as the index of α2,6 Sia binding preference.

### Modeling of the EG/D1 N1 neuraminidase structure and energetic assessment of adaptive mutations

The amino acid sequence of N1 NA (EG/D1) was retrieved from GenBank (ID: AB497032), and the structure was modeled based on a crystal structure of N1 NA (A/Vietnam/1203/04) [64] as a template (PDB: 2HTY). Based on the template, different amino acids were modeled using the rotamer library in Rosetta 3.13 [65]. The initial N1 model structure was then refined further using the FastRelax protocol with all-atom constraints [66]. Finally, the energetics (ΔΔG = ΔG_Mut_ – ΔG_WT_) of the mutations were assessed using the cartesian_ddG protocol in Rosetta [67]. The structure was visualized with ChimeraX [68].

### Statistical analysis

Data analyses were conducted using GraphPad Prism 6 (GraphPad Software). Statistically significant differences between virus pairs were determined by ANOVA with Tukey’s multiple comparison test. For pairwise comparisons (e.g., HA-only vs. HA/NA simultaneous mutants; NA amount–normalized activity analyses), two-tailed unpaired t-tests were performed.

## Supporting information

S1 Fig Fluorescent lectin staining of α2,3- and α2,6-Sia on DF-1, MDCK, untreated 1A5, and α2,3-sialidase-treated 1A5 cells. DF-1, MDCK, untreated 1A5 cells, and 1A5 cells treated with α2,3-specific sialidase were analyzed for cell-surface Sia composition using fluorescent lectin staining. Cells were stained with MAL-I (α2,3 Sia), SNA (α2,6 Sia), and Hoechst (nuclei). Representative images of individual signals (MAL-I, SNA, Hoechst) and merged channels are shown. For α2,3-sialidase-treated 1A5 cells, staining was performed immediately after treatment (0 h) and after 13 h to assess temporal stability of α2,3 Sia depletion during infection experiments. MAL-I signals remained undetectable at both time points, whereas SNA staining remained intact, confirming that α2,3 Sia were durably depleted for at least 13 h. This 13-h window matches the duration of the infection experiments (1-h virus adsorption + 12-h incubation) used in Figs 4 and 5, thereby confirming that the receptor environment remained constant during the viral replication assays. Scale bar: 25 μm.
(TIF)

S2 Fig In vitro replication kinetics of intra-patient NA mutant viruses. Viral replication kinetics of intra-patient NA mutant viruses in (A) DF-1, (B) MDCK, (C) 1A5, and (D) α2,3-sialidase-treated 1A5 cells. Viruses were inoculated as described in the Fig 4 legend, and virus yields in supernatants were quantified by FFU assay over a 72-h time course. The dotted line indicates NA-wt, and solid lines indicate NA mutant viruses. For α2,3-sialidase-treated 1A5 cells, α2,3-Sia depletion was experimentally confirmed to persist through the 13-h interval used for the main analyses (Figs 4 and 5); the extended 96-h kinetics shown here are provided as supplementary reference data.
(PDF)

S3 Fig In vitro replication kinetics of intra-cellular NA mutant viruses. Viral replication kinetics of intra-cellular NA mutant viruses in (A) DF-1, (B) MDCK, (C) 1A5, and (D) α2,3-sialidase-treated 1A5 cells. Viruses were inoculated as described in the Fig 4 legend, and virus yields in supernatants were quantified by FFU assay over a 72-h time course. The dotted line indicates NA-wt, and solid lines indicate NA mutant viruses. For α2,3-sialidase-treated 1A5 cells, α2,3-Sia depletion was experimentally confirmed to persist through the 13-h interval used in the main analyses (Figs 4 and 5); the extended 96-h kinetics presented here are provided as supplementary reference data.
(PDF)

S4 Fig Quantification of NA protein levels in purified virions and correlation with intracellular NA expression. (A, D) Representative Western blots showing NA and NP proteins in purified virus particles bearing intra-patient NA mutations (A) or intra-cellular NA mutations (D). Virus stocks were purified by ultracentrifugation, and the resulting virion preparations were normalized by FFU titers before SDS-PAGE and Western blot analysis. NA band intensities were quantified, normalized to NP in the same lane, and expressed relative to the wt virus. (B, E) Quantified amounts of NA incorporated into purified virions for intra-patient (B) and intra-cellular (E) NA mutants. Data represent mean ± SD from three independent experiments. (C, F) Correlation between intracellular NA expression and virion-associated NA levels for intra-patient (C) and intra-cellular (F) NA mutants. Intracellular expression values were obtained from the Western blot analysis shown in Fig 6, whereas virion-associated NA levels were determined as described above. Strong correlations were observed (R^2^ = 0.7256 for intra-patient mutants; R^2^ = 0.7863 for intra-cellular mutants), indicating that intracellular NA expression provides a reliable surrogate measure for NA content incorporated into virions. This dataset forms the quantitative basis for calculating NA amount–normalized (“intrinsic”) NA activity shown in S5 Fig.
(TIF)

S5 Fig NA amount–normalized (“intrinsic”) NA activity of NA mutant viruses classified by their occurrence in human infections or in human cell culture. Intrinsic NA activity was calculated by normalizing virion sialidase activity—measured from FFU-normalized virion preparations—to NA protein abundance quantified in S4 Fig. (A) NA amount–normalized activity of mutations with higher prevalence in human virus NAs. (B) NA amount–normalized activity of mutations detected only in human virus NAs or those including them. (C) NA amount–normalized activity of NA mutations selected during replication in human cells. Each data point represents the mean ± SD from three independent experiments. *P < 0.01. Overall, these analyses demonstrate that human-prevalent NA mutations generally retain intrinsic enzymatic activity, whereas mutations detected exclusively in human isolates or selected in human cells frequently show substantial intrinsic activity loss. This distinction highlights which mutation categories reduce virion-level (“net”) NA activity via decreased NA incorporation versus via reduced intrinsic catalytic efficiency.
(TIF)

S1 Table Co-occurrence of HA-adaptive and NA-adaptive mutations in Egyptian clade 2.2.1 H5N1 clinical isolates. (PDF)

S2 Table Unique HA/NA adaptive mutation combinations identified in Egyptian clade 2.2.1 H5N1 clinical isolates. (PDF)

S1 Data Source Data. (XLSX)
