# Supplementary figures and images for: Intra-patient neuraminidase mutations in avian H5N1 influenza virus reduce sialidase activity to complement weaker hemagglutinin binding and facilitate human infection
Source: PLoS Pathog. 2026 Jan 23;22(1):e1013863. doi: 10.1371/journal.ppat.1013863 (PMC12829795; doi:10.1371/journal.ppat.1013863)

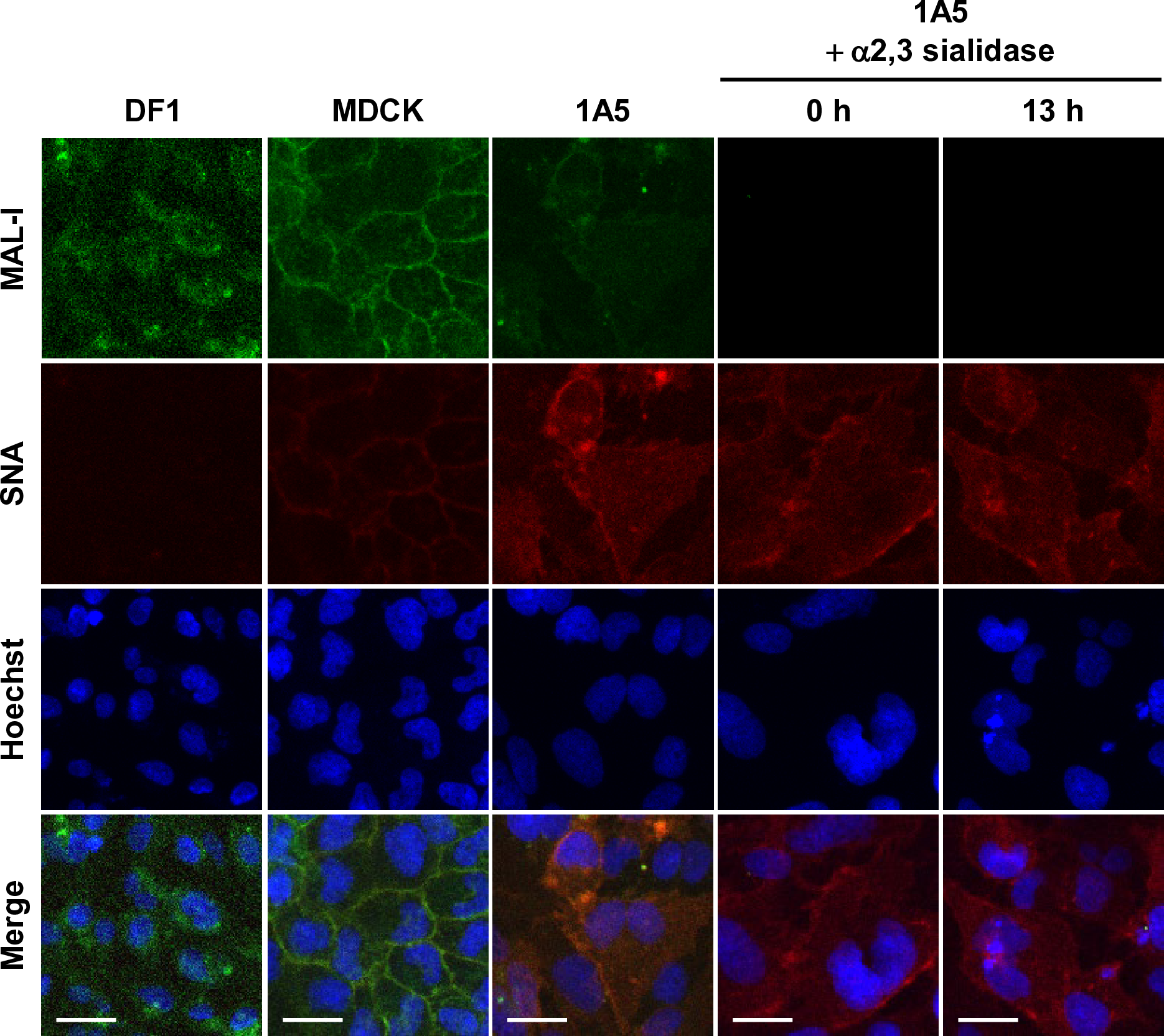

Supplement: S1 Fig — DF-1, MDCK, untreated 1A5 cells, and 1A5 cells treated with α2,3-specific sialidase were analyzed for cell-surface Sia composition using fluorescent lectin staining. Cells were stained with MAL-I (α2,3 Sia), SNA (α2,6 Sia), and Hoechst (nuclei). Representative images of individual signals (MAL-I, SNA, Hoechst) and merged channels are shown. For α2,3-sialidase-treated 1A5 cells, staining was performed immediately after treatment (0 h) and after 13 h to assess temporal stability of α2,3 Sia depletion during infection experiments. MAL-I signals remained undetectable at both time points, whereas SNA staining remained intact, confirming that α2,3 Sia were durably depleted for at least 13 h. This 13-h window matches the duration of the infection experiments (1-h virus adsorption + 12-h incubation) used in Figs 4 and 5, thereby confirming that the receptor environment remained constant during the viral replication assays. Scale bar: 25 μm. (TIF) [file ppat.1013863.s001.tif]

Intra-patient NA mutations

A DF-1

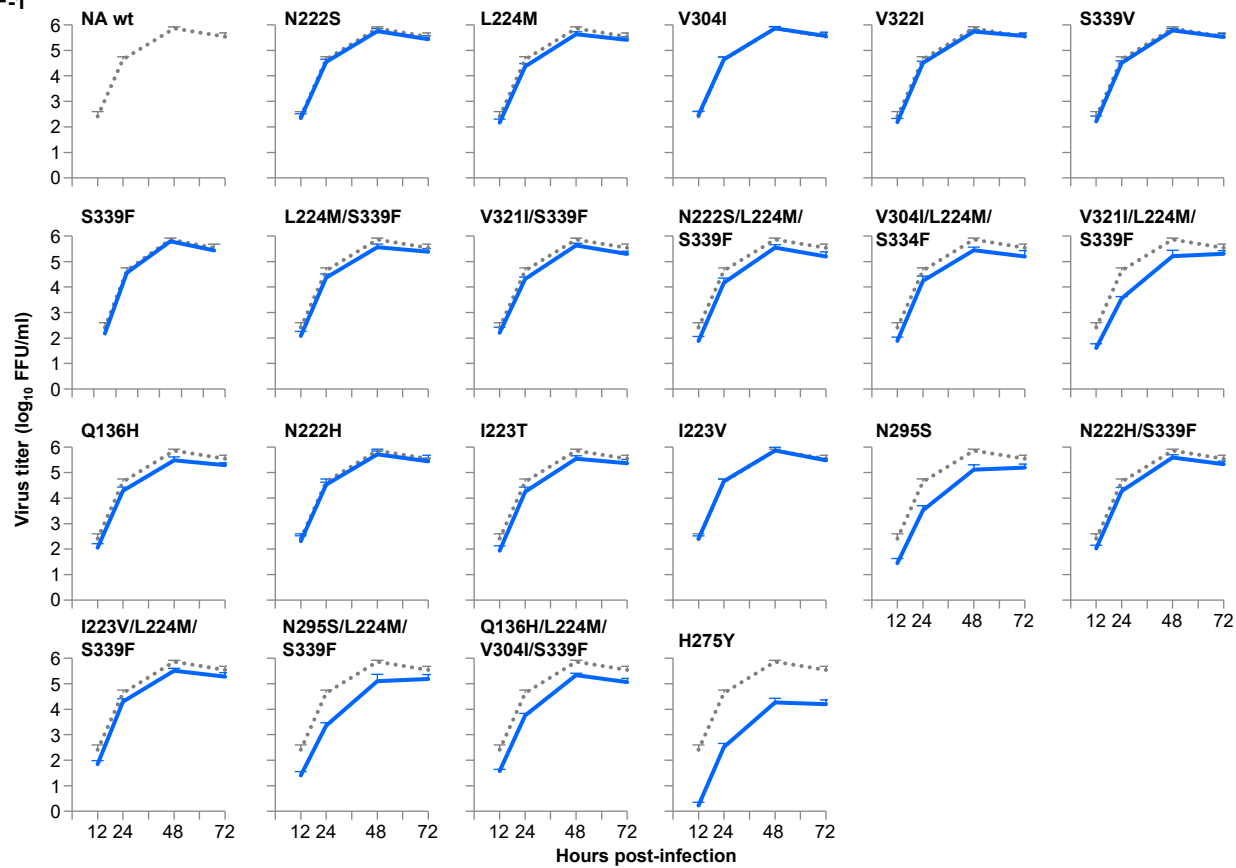

B MDCK

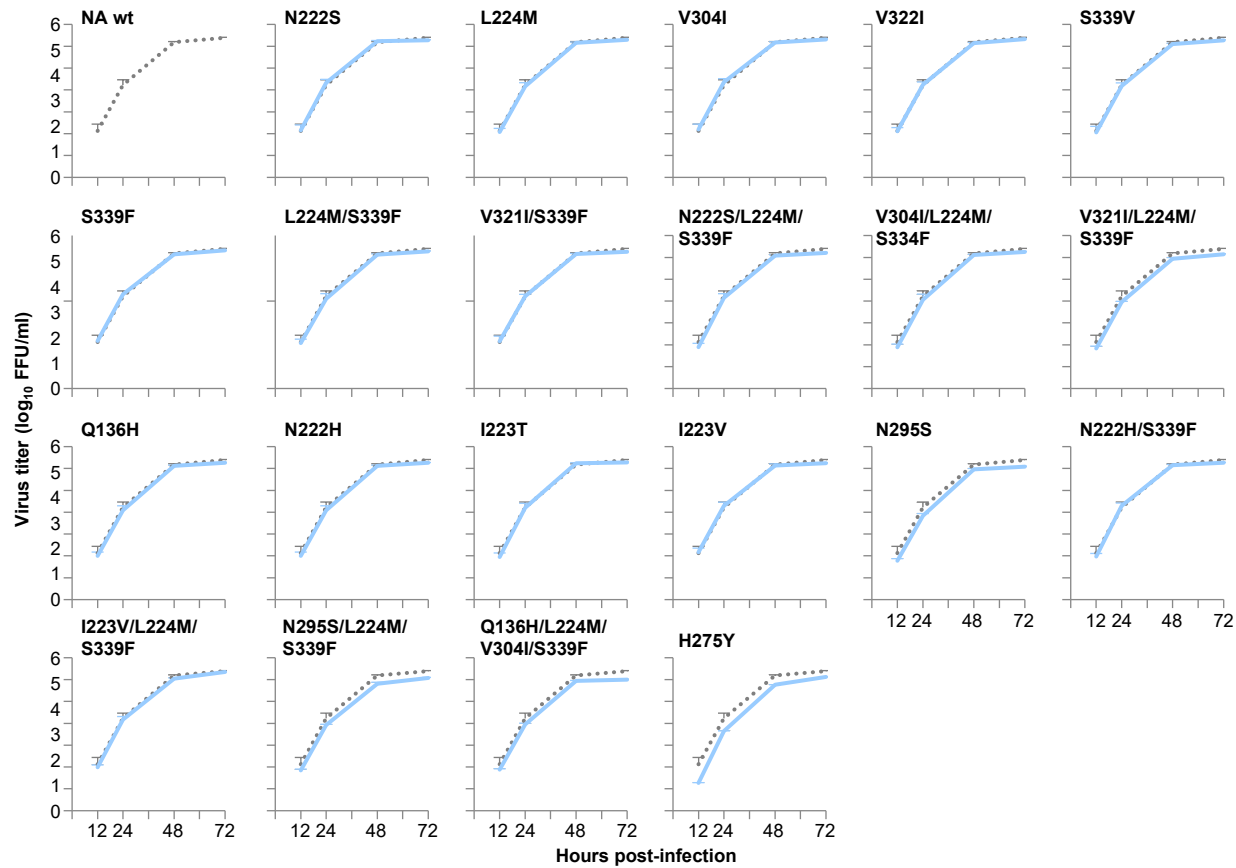

Intra-patient NA mutations

C 1A5

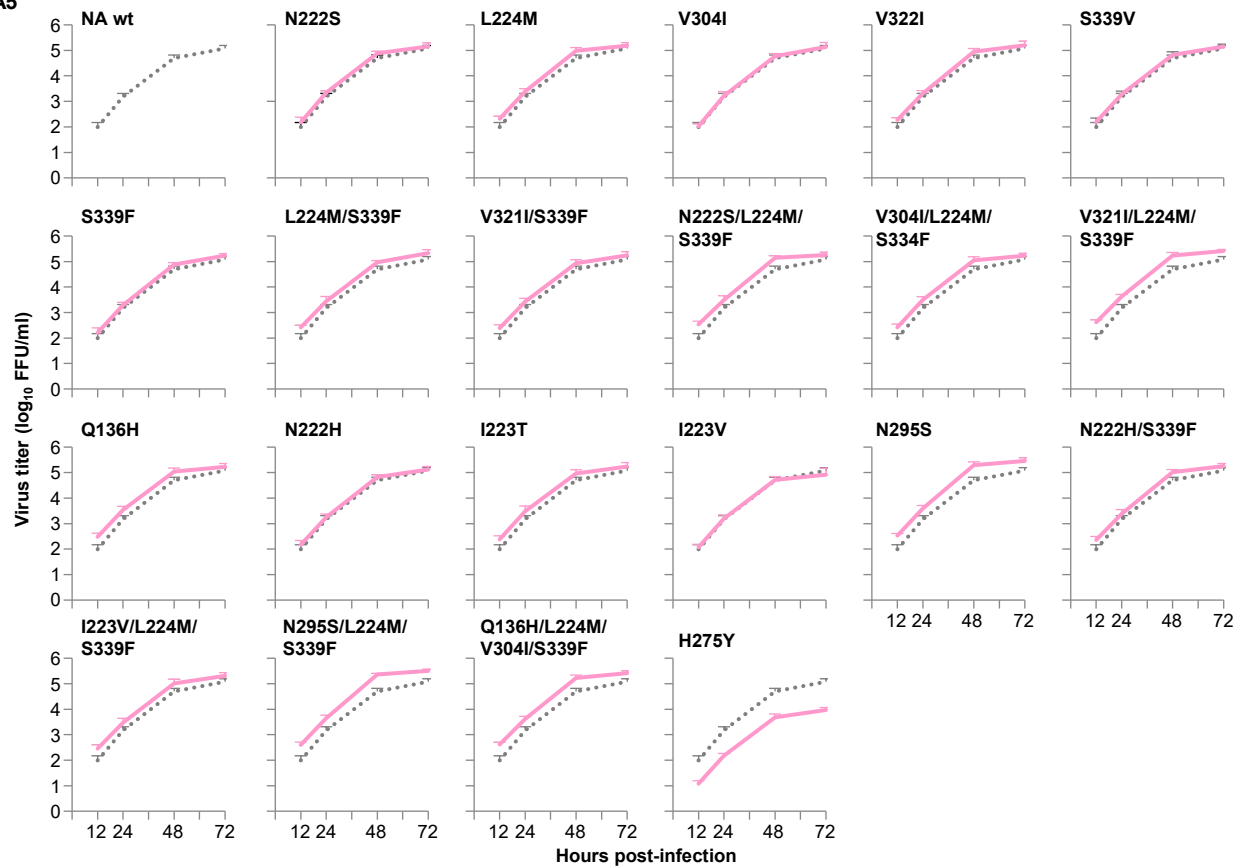

D  $\alpha$ 2,3 sialidase-treated 1A5

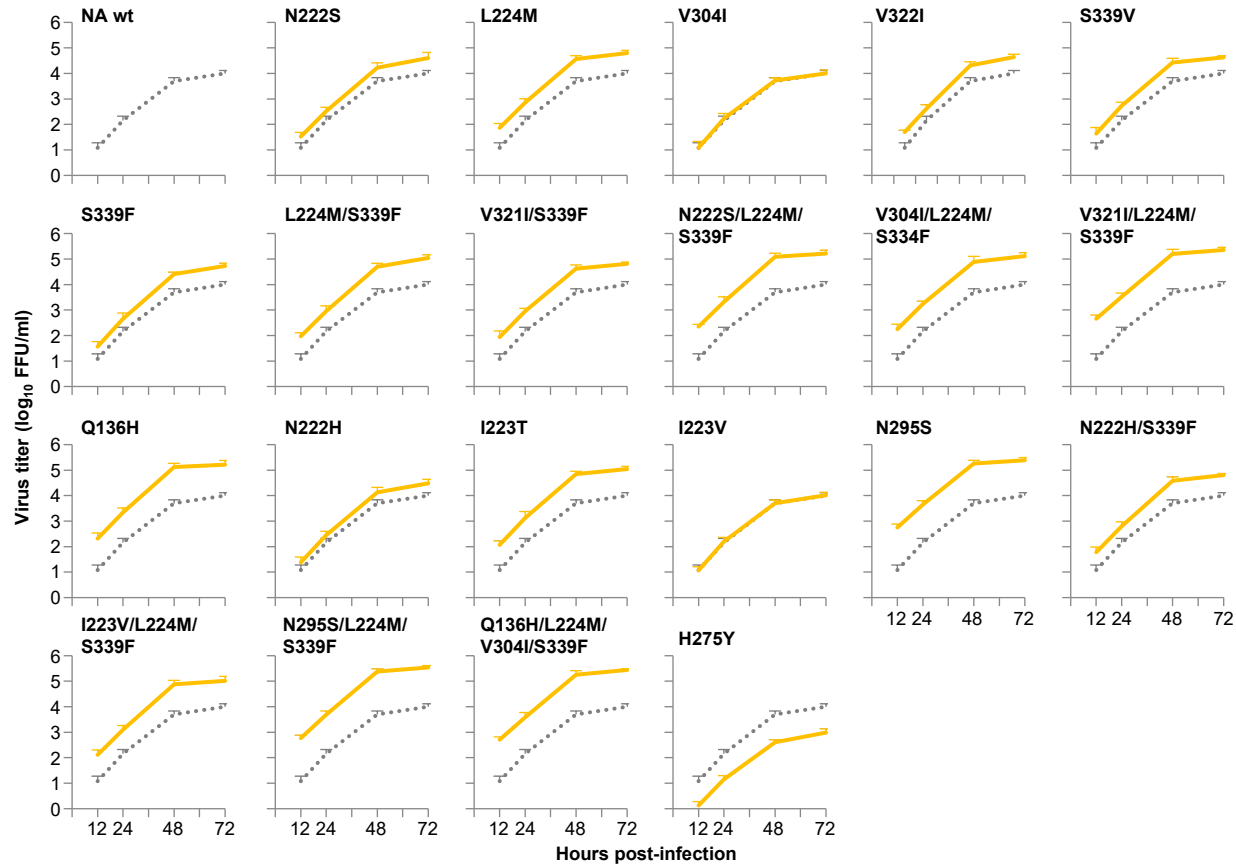

Supplement: S2 Fig — Viral replication kinetics of intra-patient NA mutant viruses in (A) DF-1, (B) MDCK, (C) 1A5, and (D) α2,3-sialidase-treated 1A5 cells. Viruses were inoculated as described in the Fig 4 legend, and virus yields in supernatants were quantified by FFU assay over a 72-h time course. The dotted line indicates NA-wt, and solid lines indicate NA mutant viruses. For α2,3-sialidase-treated 1A5 cells, α2,3-Sia depletion was experimentally confirmed to persist through the 13-h interval used for the main analyses (Figs 4 and 5); the extended 96-h kinetics shown here are provided as supplementary reference data. (PDF) [file ppat.1013863.s002.pdf]

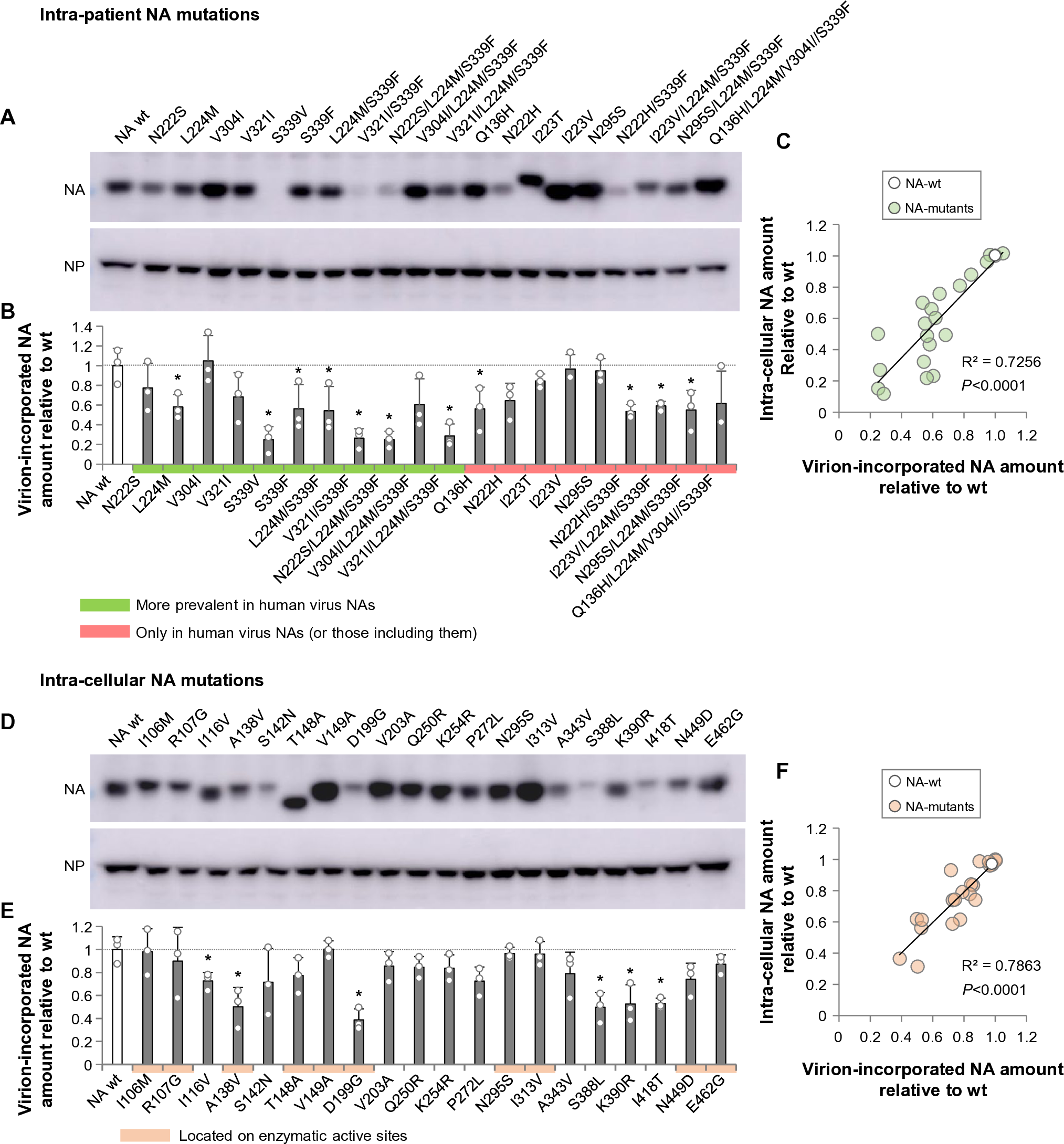

Supplement: S4 Fig — (A, D) Representative Western blots showing NA and NP proteins in purified virus particles bearing intra-patient NA mutations (A) or intra-cellular NA mutations (D). Virus stocks were purified by ultracentrifugation, and the resulting virion preparations were normalized by FFU titers before SDS-PAGE and Western blot analysis. NA band intensities were quantified, normalized to NP in the same lane, and expressed relative to the wt virus. (B, E) Quantified amounts of NA incorporated into purified virions for intra-patient (B) and intra-cellular (E) NA mutants. Data represent mean ± SD from three independent experiments. (C, F) Correlation between intracellular NA expression and virion-associated NA levels for intra-patient (C) and intra-cellular (F) NA mutants. Intracellular expression values were obtained from the Western blot analysis shown in Fig 6, whereas virion-associated NA levels were determined as described above. Strong correlations were observed (R2 = 0.7256 for intra-patient mutants; R2 = 0.7863 for intra-cellular mutants), indicating that intracellular NA expression provides a reliable surrogate measure for NA content incorporated into virions. This dataset forms the quantitative basis for calculating NA amount–normalized (“intrinsic”) NA activity shown in S5 Fig. (TIF) [file ppat.1013863.s004.tif]

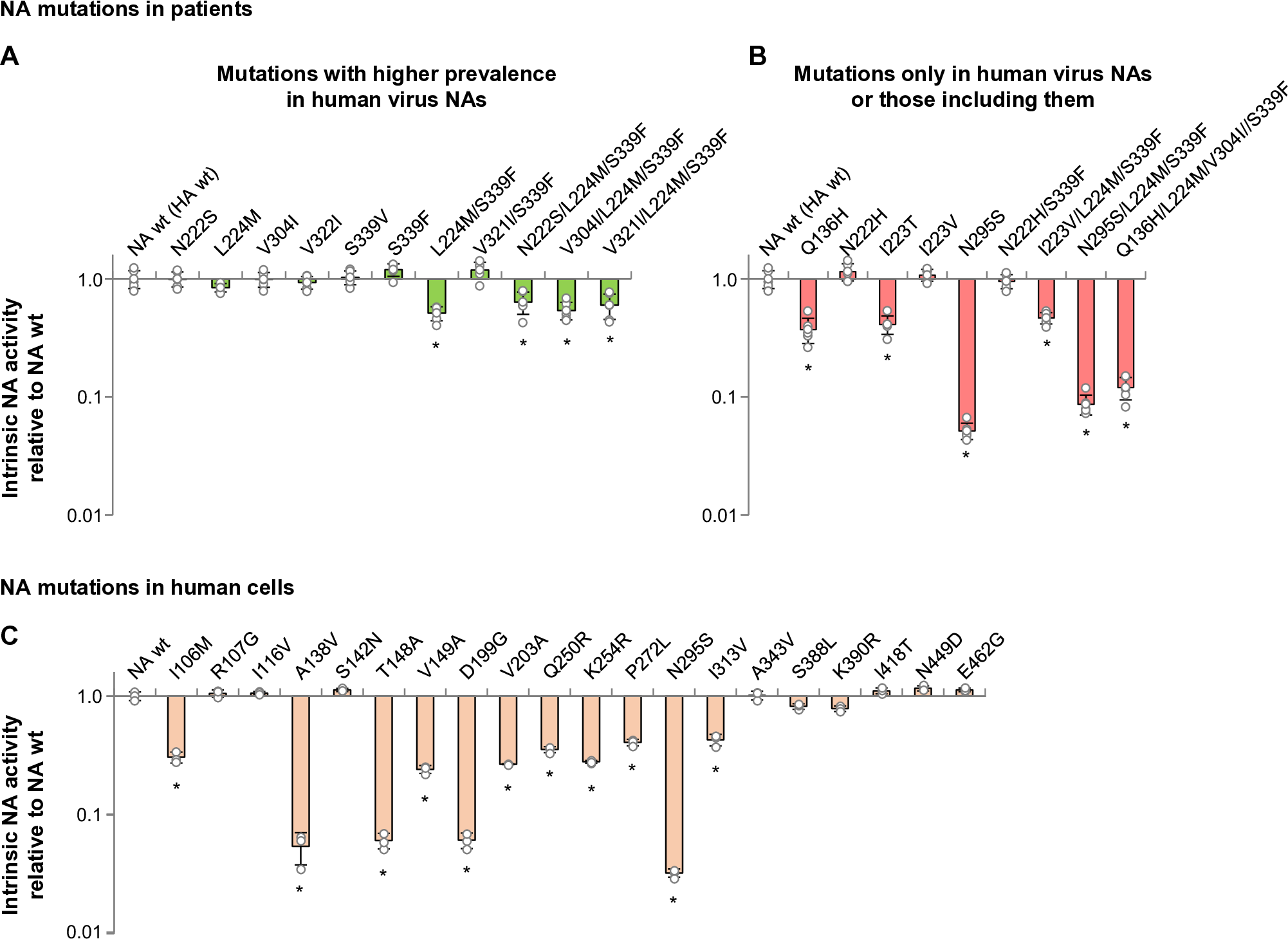

Supplement: S5 Fig — Intrinsic NA activity was calculated by normalizing virion sialidase activity—measured from FFU-normalized virion preparations—to NA protein abundance quantified in S4 Fig. (A) NA amount–normalized activity of mutations with higher prevalence in human virus NAs. (B) NA amount–normalized activity of mutations detected only in human virus NAs or those including them. (C) NA amount–normalized activity of NA mutations selected during replication in human cells. Each data point represents the mean ± SD from three independent experiments. *P < 0.01. Overall, these analyses demonstrate that human-prevalent NA mutations generally retain intrinsic enzymatic activity, whereas mutations detected exclusively in human isolates or selected in human cells frequently show substantial intrinsic activity loss. This distinction highlights which mutation categories reduce virion-level (“net”) NA activity via decreased NA incorporation versus via reduced intrinsic catalytic efficiency. (TIF) [file ppat.1013863.s005.tif]
