# Supplementary material for: Intra-patient neuraminidase mutations in avian H5N1 influenza virus reduce sialidase activity to complement weaker hemagglutinin binding and facilitate human infection
Source: PLoS Pathog. 2026 Jan 23;22(1):e1013863. doi: 10.1371/journal.ppat.1013863 (PMC12829795; doi:10.1371/journal.ppat.1013863)

Intra-cellular NA mutations

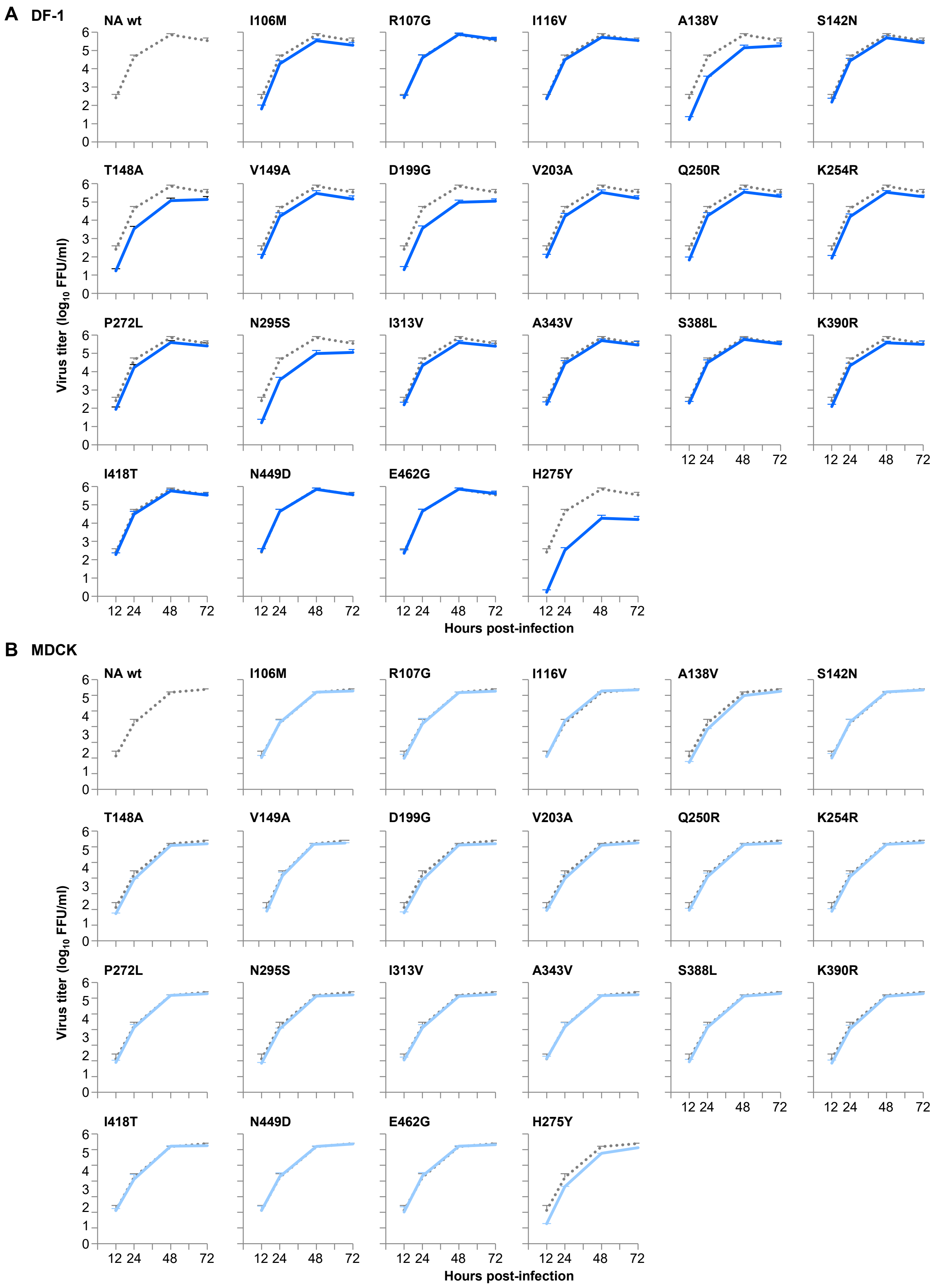

Intra-cellular NA mutations

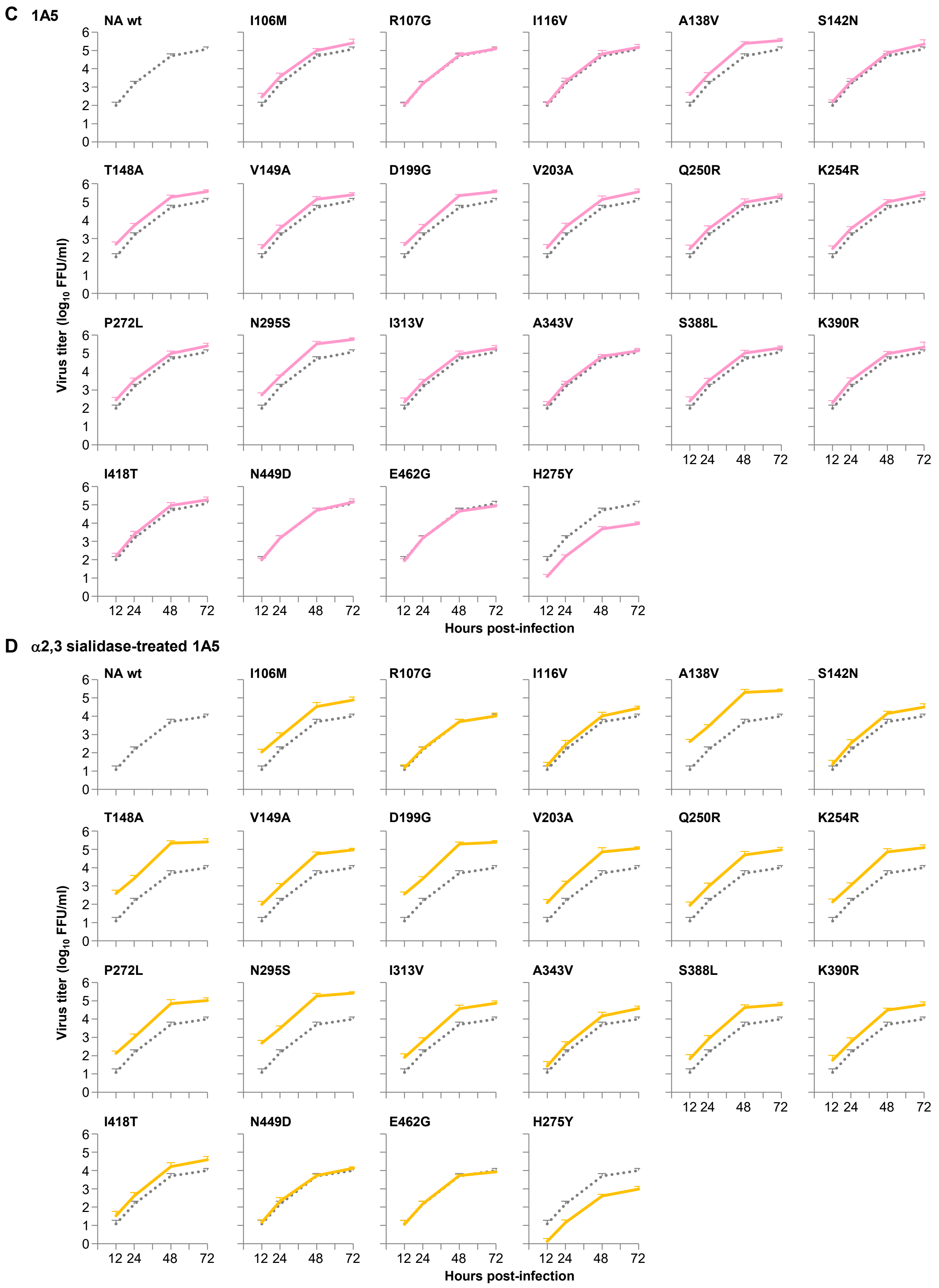

Supplement: S3 Fig — Viral replication kinetics of intra-cellular NA mutant viruses in (A) DF-1, (B) MDCK, (C) 1A5, and (D) α2,3-sialidase-treated 1A5 cells. Viruses were inoculated as described in the Fig 4 legend, and virus yields in supernatants were quantified by FFU assay over a 72-h time course. The dotted line indicates NA-wt, and solid lines indicate NA mutant viruses. For α2,3-sialidase-treated 1A5 cells, α2,3-Sia depletion was experimentally confirmed to persist through the 13-h interval used in the main analyses (Figs 4 and 5); the extended 96-h kinetics presented here are provided as supplementary reference data. (PDF) [file ppat.1013863.s003.pdf]
