# Supplementary material for: Intra-patient neuraminidase mutations in avian H5N1 influenza virus reduce sialidase activity to complement weaker hemagglutinin binding and facilitate human infection
Source: PLoS Pathog. 2026 Jan 23;22(1):e1013863. doi: 10.1371/journal.ppat.1013863 (PMC12829795; doi:10.1371/journal.ppat.1013863)
Supplement: S1 Table — (PDF) [file ppat.1013863.s006.pdf]

S1 Table. Co-occurrence of HA-adaptive and NA-adaptive mutations in Egyptian clade 2.2.1 H5N1 clinical isolates*

Serial no.	Virus Strain	Subtype	Passage History[†]	Collection Date	Host age (yr)	Host gender	HA adaptive mutations	NA adaptive mutations
1	A/Egypt/2763-NAMRU3/2006	H5N1		2006-03-16	30	Female	D154N/K28R	
2	A/Egypt/2782-NAMRU3/2006	H5N1		2006-10	30	Male		
3	A/Egypt/2783-NAMRU3/2006	H5N1		2006	*U*[‡]	*U*[‡]		
4	A/Egypt/2786-NAMRU3/2006	H5N1		2006	*U*[‡]	*U*[‡]		
5	A/Egypt/2947-NAMRU3/2006	H5N1		2006	18	Female	S223N	
6	A/Egypt/2991-NAMRU3/2006	H5N1		2006-04-01	1	Female		S338F
7	A/Egypt/2992-NAMRU3/2006	H5N1		2006-04-01	6	Female		S338F
8	A/Egypt/3105-NAMRU3/2006	H5N1		2006-04-03	16	Female		
9	A/Egypt/3458-NAMRU3/2006	H5N1		2006	16	Female		
10	A/Egypt/5494-NAMRU3/2006	H5N1		2006	18	Female		
11	A/Egypt/5614-NAMRU3/2006	H5N1		2006-05-02	27	Female	K28R	
12	A/Egypt/7021-NAMRU3/2006	H5N1		2006-05-16	75	Female		
13	A/Egypt/902782/2006	H5N1		2006	*U*[‡]	*U*[‡]		
14	A/Egypt/902786/2006	H5N1		2006	*U*[‡]	*U*[‡]		
15	A/Egypt/12374-NAMRU3/2006	H5N1		2006-10	39	Female		
16	A/Egypt/14724-NAMRU3/2006	H5N1		2006	26	Male	A127T	
17	A/Egypt/14725-NAMRU3/2006	H5N1		2006	15	Female	A127T	
18	A/Egypt/0636-NAMRU3/2007	H5N1		2007	27	Female		S338F
19	A/Egypt/1394-NAMRU3/2007	H5N1		2007-02-05	17	Female		S338F
20	A/Egypt/1604-NAMRU3/2007	H5N1		2007-02-12	37	Female		
21	A/Egypt/1731-NAMRU3/2007	H5N1		2007-02-14	5	Male		
22	A/Egypt/1902-NAMRU3/2007	H5N1		2007-02-28	4	Female	D154N	
23	A/Egypt/2256-NAMRU3/2007	H5N1		2007-03-10	4	Male		
24	A/Egypt/2321-NAMRU3/2007	H5N1		2007-03-13	10	Male	H125Y/D154N/T188I/N94D	S338F
25	A/Egypt/2331-NAMRU3/2007	H5N1		2007-03-16	2	Male	H125Y/D154N/T188I/N94D	S338F
26	A/Egypt/2616-NAMRU3/2007	H5N1		2007-03-24	3	Female	H125Y/D154N/T188I/N94D	S338F
27	A/Egypt/2620-NAMRU3/2007	H5N1		2007-03-25	5	Male	H125Y/D154N/N94D	S338F
28	A/Egypt/2621-NAMRU3/2007	H5N1		2007-03-25	6	Male		L223M/S338F
29	A/Egypt/2629-NAMRU3/2007	H5N1		2007-03-29	7	Male		L223M/S338F
30	A/Egypt/2630-NAMRU3/2007	H5N1		2007-03-30	4	Male		
31	A/Egypt/2631-NAMRU3/2007	H5N1		2007-03-30	4	Female		S338F
32	A/Egypt/2750-NAMRU3/2007	H5N1		2007-04-04	2	Female	N94D	N221H/S338F
33	A/Egypt/2751-NAMRU3/2007	H5N1		2007-04-06	15	Female	A184E	
34	A/Egypt/4081-NAMRU3/2007	H5N1		2007-06-07	10	Female	N94D	S338F
35	A/Egypt/4082-NAMRU3/2007	H5N1		2007-06-10	4	Female	N94D	S338F
36	A/Egypt/4226-NAMRU3/2007	H5N1		2007-06-21	4	Female	N94D	S338F
37	A/Egypt/6251-NAMRU3/2007	H5N1		2007-07-21	25	Female	D154G	
38	A/Egypt/10211-NAMRU3/2007	H5N1		2007-12-24	25	Female	N94D	S338F
39	A/Egypt/10215-NAMRU3/2007	H5N1		2007-12-26	22	Female		
40	A/Egypt/10216-NAMRU3/2007	H5N1		2007-12-29	36	Female		
41	A/Egypt/10217-NAMRU3/2007	H5N1		2007-12-29	30	Female	S223N/D154G	
42	A/Egypt/1980-NAMRU3/2008	H5N1		2008-02-26	4	Female	D154E	S338F
43	A/Egypt/2289-NAMRU3/2008	H5N1		2008-03-02	25	Female	S223I/128∆/I151T	S338F
44	A/Egypt/2514-NAMRU3/2008	H5N1		2008-03-04	11	Male	N182K/T195I/N94D	S338F
45	A/Egypt/2546-NAMRU3/2008	H5N1		2008-03-08	8	Male	S223N	S338F
46	A/Egypt/3158-NAMRU3/2008	H5N1		2008-04-05	19	Male	N94D	S338F
47	A/Egypt/3300-NAMRU3/2008	H5N1		2008-04-11	30	Female	A184E/D154N	
48	A/Egypt/3401-NAMRU3/2008	H5N1		2008-04-16	2	Male	D154G	
49	A/Egypt/N00001/2009	H5N1	E1	2009-01-11	1	Female		L223M/S338F
50	A/Egypt/N00585/2009	H5N1		2009-01-23	2	Female	D154N/128∆/I151T	L223M/S338F
51	A/Egypt/N00605/2009	H5N1		2009-02-03	2	Male	D154N	L223M/S338F
52	A/Egypt/N00606/2009	H5N1	E2	2009-02-07	1	Male	D154N	V320I/S338F
53	A/Egypt/N01310/2009	H5N1		2009-02-28	2	Male	128∆/I151T	S338F
54	A/Egypt/N02039/2009	H5N1		2009-03-03	2	Male	V131M/128∆/I151T	L223M/S338F
55	A/Egypt/N02407/2009	H5N1	E1	2009-03-09	1	Female	128∆/I151T	L223M/S338F
56	A/Egypt/N02563/2009	H5N1	C1	2009-03-14	38	Female	A185E/D154N/128∆/I151T	L223M/S338F
57	A/Egypt/N02752/2009	H5N1	C1	2009-03-24	2	Female	128∆/I151T	V320I/L223M/S338F
58	A/Egypt/N03228/2009	H5N1	C1	2009-03-30	2	Male	S223N/128∆/I151T	L223M/S338F
59	A/Egypt/N03272/2009	H5N1	C1	2009-04-01	1	Male	128∆/I151T	L223M/S338F
60	A/Egypt/N03434/2009	H5N1		2009-04-15	33	Female	Q192H/N94D	S338V
61	A/Egypt/N03438/2009	H5N1		2009-04-16	25	Female		
62	A/Egypt/N03439/2009	H5N1	E1	2009-04-18	1	Female	128∆/I151T	L223M/S338F
63	A/Egypt/N03450/2009	H5N1	E1	2009-04-19	4	Male	A185T/D154N/128∆/I151T	L223M/S338F
64	A/Egypt/N04316/2009	H5N1		2009-05-09	5	Male	A185E/D154N/128∆/I151T	L223M/S338F
65	A/Egypt/N04394/2009	H5N1	E1	2009-05-11	3	Male	128∆/I151T	I222V/L223M/S338F
66	A/Egypt/N04395/2009	H5N1	E1	2009-05-13	3	Male	128∆/I151T	L223M/S338F
67	A/Egypt/N04396/2009	H5N1	E1	2009-05-17	4	Female	A134S/128∆/I151T	L223M/S338F
68	A/Egypt/N04526/2009	H5N1	C1	2009-05-18	4	Male	Q192H/N94D	S338V
69	A/Egypt/N04527/2009	H5N1	C1	2009-05-18	*U*[‡]	*U*[‡]	A185T/128∆/I151T	V320I/L223M/S338F
70	A/Egypt/N04822/2009	H5N1	E1	2009-05-25	4	Female	Q192H/N94D	S338V
71	A/Egypt/N04823/2009	H5N1	E1	2009-05-25	4	Female	D154N/128∆/I151T	L223M/S338F
72	A/Egypt/N04830/2009	H5N1		2009-05-29	1	Female	128∆/I151T	L223M/S338F
73	A/Egypt/N04979/2009	H5N1	C1	2009-05-31	4	Female	Q192H/N94D	
74	A/Egypt/N05056/2009	H5N1	C1	2009-06-06	4	Female	K152Q/128∆/I151T/K22R	Q136H/V320I/V303I/L223M
75	A/Egypt/N05912/2009	H5N1		2009-06-16	1	Male	A184G/D154N/128∆/I151T/Q15H	L223M/S338F
76	A/Egypt/N07392/2009	H5N1		2009-07-25	8	Female	D154N/128∆/I151T	L223M/S338F
77	A/Egypt/N07908/2009	H5N1	E1	2009-07-30	1	Male	A184G/D154N/128∆/I151T/Q15H	L223M/S338F
78	A/Egypt/N08835/2009	H5N1		2009-08-01	<1	Female	R189G/D154N/128∆/I151T	L223M/S338F
79	A/Egypt/N09174/2009	H5N1	E1	2009-08-26	1	Female	D154N/128∆/I151T	L223M/S338F
80	A/Egypt/N09539/2009	H5N1	E1	2009-09-16	13	Male	D154N/128∆/I151T	L223M/S338F
81	A/Egypt/N11981/2009	H5N1	E2	2009-11-22	3	Male	D154N/128∆/I151T	N294S/L223M/S338F
82	A/Egypt/N15262/2009	H5N1	E2	2009-12	3	Male	A185E/D154N/128∆/I151T	L223M/S338F
83	A/Egypt/N00269/2010	H5N1	E2	2010-01-11	20	Female	A185T/128∆/I151T	N221S/L223M/S338F
84	A/Egypt/N00270/2010	H5N1	E2	2010-01-12	1	Male	D154N/128∆/I151T	
85	A/Egypt/N01360/2010	H5N1	C1	2010-02-02	40	Female	R140K/128∆/I151T	L223M/S338F
86	A/Egypt/N01644/2010	H5N1	E1	2010-02-07	37	Male	A134V/128∆/I151T	V303I/L223M/S338F
87	A/Egypt/N01982/2010	H5N1	E1	2010-02-12	30	Female	D154N/128∆/I151T	L223M/S338F
88	A/Egypt/N02038/2010	H5N1	E2	2010-02-15	10	Male	K152Q/D154N/128∆/I151T	L223M/S338F
89	A/Egypt/N02127/2010	H5N1	E1	2010-02-13	30	Female	D154N/128∆/I151T	L223M/S338F
90	A/Egypt/N02554/2010	H5N1	E1	2010-02-23	1	Male	R189S/D154N/A184E/128∆/I151T	L223M/S338F
91	A/Egypt/N02770/2010	H5N1		2010-02-27	53	Male	D154N/128∆/I151T/K48E	V303I/L223M/S338F
92	A/Egypt/N03071/2010	H5N1	E1	2010-03-03	1	Male	A184E/D154N/128∆/I151T	L223M/S338F
93	A/Egypt/N03072/2010	H5N1	E1	2010-03-07	20	Female	128∆/I151T	V303I/L223M/S338F
94	A/Egypt/N04434/2010	H5N1		2010-03-31	18	Female	128∆/I151T/K35R	V303I/L223M/S338F

*Information were collected from NBCI IVR and GISAID (http://platform.gisaid.org/).

[†]C, culured cells; E, eggs

[‡]*U* , unknown
