# Supplementary material for: Intra-patient neuraminidase mutations in avian H5N1 influenza virus reduce sialidase activity to complement weaker hemagglutinin binding and facilitate human infection
Source: PLoS Pathog. 2026 Jan 23;22(1):e1013863. doi: 10.1371/journal.ppat.1013863 (PMC12829795; doi:10.1371/journal.ppat.1013863)
Supplement: S2 Table — (PDF) [file ppat.1013863.s007.pdf]

S2 Table. Unique HA/NA adaptive mutation combinations identified in Egyptian clade 2.2.1 H5N1 clinical isolates

Serial no.	HA mutations	NA mutations	no. of human strains
1	N94D	S338F	5
2	D154E	S338F	1
3	S223N	S338F	1
4	128∆/I151T	S338F	1
5	H125Y/D154N/N94D	S338F	1
6	H125Y/D154N/T188I/N94D	S338F	3
7	N182K/T195I/N94D	S338F	1
8	S223I/128∆/I151T	S338F	1
9	Q192H/N94D	S338V	3
10	N94D	N221H/S338F	1
11	D154N	L223M/S338F	1
12	128∆/I151T	L223M/S338F	5
13	V131M/128∆/I151T	L223M/S338F	1
14	A134S/128∆/I151T	L223M/S338F	1
15	R140K/128∆/I151T	L223M/S338F	1
16	K152Q/D154N/128∆/I151T	L223M/S338F	1
17	D154N/128∆/I151T	L223M/S338F	7
18	A184E/D154N/128∆/I151T	L223M/S338F	1
19	A184G/D154N/128∆/I151T/Q15H	L223M/S338F	2
20	A185E/D154N/128∆/I151T	L223M/S338F	3
21	A185T/D154N/128∆/I151T	L223M/S338F	1
22	R189G/D154N/128∆/I151T	L223M/S338F	1
23	R189S/D154N/A184E/128∆/I151T	L223M/S338F	1
24	S223N/128∆/I151T	L223M/S338F	1
25	D154N	V320I/S338F	1
26	A185T/128∆/I151T	N221S/L223M/S338F	1
27	128∆/I151T	I222V/L223M/S338F	1
28	D154N/128∆/I151T	N294S/L223M/S338F	1
29	128∆/I151T	V303I/L223M/S338F	1
30	128∆/I151T/K35R	V303I/L223M/S338F	1
31	A134V/128∆/I151T	V303I/L223M/S338F	1
32	D154N/128∆/I151T	V303I/L223M/S338F	1
33	128∆/I151T	V320I/L223M/S338F	1
34	A185T/128∆/I151T	V320I/L223M/S338F	1
35	K152Q/128∆/I151T/K22R	Q136H/V320I/V303I/L223M	1
		total	56
